# Synthesis of facile polystyrene/pol(N-methylaniline)/CuO composite for methyl orange uptake

**DOI:** 10.1038/s41598-026-63266-9

**Published:** 2026-08-05

**Authors:** Aya H. M. Abd El-Gied, A. B. Khaliel, H. M. Abd El-Salam

**Affiliations:** Department of Chemistry, Faculty of Science, Polymer Research Laboratory, Beni-Suef University, Beni-Suef City, 62514 Egypt

**Keywords:** Polystyrene/poly(N-methylaniline), Polystyrene/poly(N-methylaniline)/CuO, Methyl orange removal, Adsorption isotherm, Kinetic study, Optimum condition, Chemistry, Environmental sciences, Materials science

## Abstract

Polystyrene (PS) was modified by grafting with poly(N-methylaniline) (PNMANI) and assembling on CuO nanoparticles to be a good adsorbent to methyl orange (MO). Dye-contaminated wastewater remains a critical environmental concern, requiring efficient and economically viable treatment strategies. Herein, polystyrene/poly(N-methylaniline) (PS/PNMANI) and polystyrene/ poly(N-methylaniline)/ CuO (PS/PNMANI/CuO) composite which not prepared before, were synthesized and characterized for MO uptake from aqueous media. Both structural and surface properties were analyzed using SEM, XRD, FTIR, BET, and TGA analyses. Batch adsorption experiments demonstrated that PS/PNMANI and PS/PNMANI/CuO composite achieved a maximum uptake efficiency of 95, 100% at pH 7 with an equilibrium time of 60 min using 0.10 and 0.25 g of composite respectively. The adsorption kinetics obey the pseudo-second-order model (R² = 0.997), while equilibrium data indicated mixed adsorption behavior with a maximum adsorption capacity (Qmax) of 43.29 mg/g for PS/PNMANI/CuO and 7.17 mg/g for PS/PNMANI. BET analysis revealed that the surface area of PS/PNMANI is 33.91 m²/g and 42.28 m²/g for PS/PNMANI/CuO, confirming that CuO incorporation enhanced surface characteristics and removal efficacy of graft. The improved adsorption performance is attributed to increased surface activity, synergistic polymer–CuO interactions, and porous morphology facilitating dye diffusion.

**Supplementary Information:**

The online version contains supplementary material available at 10.1038/s41598-026-63266-9.

## Introduction

The increase in technology, industrialization, and urban growth has significantly affected the cleanliness of water bodies globally^1^. Water pollution is a critical worldwide challenge, with industries playing a major role, responsible for 17–20% of the issue^2^. While dyes significantly contribute to the visual appeal of many products, their release during dyeing which has fixing efficiency between 60% and 90% presents a major environmental threat^3^. Approximately 280,000 tons of dyes are believed to be released into the environment each year, creating significant dangers to global aquatic ecosystems and water quality^4^. The advancement of synthetic dyes since the initial one was discovered in 1856, alongside the swift industrialization of textile manufacturing, has resulted in the discharge of hazardous textile dye effluents, negatively affecting aquatic ecosystems^5^. These synthetic dyes are not only harmful and diminish the dissolved oxygen levels in water, but also contribute to aesthetic issues, taking into account environmental implications and legal regulations. Eliminating dye-waste from wastewater is currently a top priority for the textile and related industries.

Cationic dyes possess higher toxicity compared to anionic dyes, and their coloring properties are significantly elevated at 1 ppm^6,7^. Cationic dyes readily engage with negatively charged cell membrane surfaces, penetrate cells, and accumulate in the cytoplasm^8^.

Methyl orange [(MO) dimethyl amino azobenzene sulfonate] is a widely used azo anionic dye. This synthetic organic dye, which is soluble in water, has a very intense orange hue, is extremely toxic and carcinogenic^9,10^, and poses risks to the environment and living organisms^11,12^. In this study, MO was chosen as a simulated pollutant, and wastewater containing MO can result in degraded water quality^13^.

The objective of this study is to eliminate MO from the aquatic environment or to enable its secondary utilization^14,15^. Different techniques, including coagulation and flocculation, oxidative breakdown, flotation, photocatalytic processes, electrochemical methods, and adsorption, have been utilized for the efficient removal of pollutants from water. Similar to all these methods, the last technique stands out as the most advantageous due to its simplicity and efficiency, attributed to its convenience, practicality, affordability, superior performance, high effectiveness, minimal sludge production, and rapidity^16–18^.

Polyaniline (PANI) and its derivatives have been thoroughly investigated for their stability in the conducting state^19–21^, facile synthesis, cost effectiveness, and distinctive redox characteristics linked to chain nitrogen^22–28^. This material is advantageous for uses in gas detection^29–32^, photovoltaic cells^33,34^, shielding from electromagnetic interference^35,36^, antistatic films^37^, and organic light-emitting diodes^38–40^.

Various configurations of polyaniline^41^ ranging from its doped and salt forms to composites and coatings on solid substrates have demonstrated significant potential as adsorbents for organic compounds and dyes. Specifically, the emeraldine salt form of PANI is highly effective for methyl orange removal due to its abundant imine and amine functional groups^42,43^. Furthermore, recent studies have highlighted the use of PANI nanoforms, such as nanoparticles and nanotubes, as highly efficient uptake of methylene blue from water^44,45^.

The widespread application of polymer composites in dye remediation processes is attributed to their superior mechanical properties, thermal resilience, and high surface-to-volume ratios^46^. Many composite materials have been applied for uptake of dyes from aqueous water solutions and wastewater. For instance, a polyamide–weathered basalt nanocomposite has been employed as an unconventional membrane for the efficient elimination of both basic and acidic dyes^47^. A polyaniline–bismuth tungstate composite has been reported to effectively remove Congo dye^48^. Additionally, polyaniline/silver nanocomposites have been utilized for the adsorption of brilliant green dye^49^, whereas polyaniline-coated gold–aryl nanoparticles have been applied for the removal of methylene blue dye^50^.

PNMANI is conductive polymer distinguished by high absorption coefficients in visible region, an extended conjugated structure^51,52^, and notable antibacterial activity^53,54^.It is also environmentally stable and suitable for wastewater treatment due to its chemical reactions that selectively adsorb dyes^55,56^. Polystyrene (PS) is a linear thermoplastic resin commonly found in everyday items due to its inexpensive production, lightweight properties, lack of odor, colorless appearance, and excellent adsorption capabilities. It is primarily marketed as a durable and thermally stable substance^57^. PS is utilized across numerous sectors, such as food packaging, medical devices, automobiles, and electrical and electronic products. The chemical composition of PS features phenyl rings and C–C single bonds, which result in a polymer that is very stable and resistant to UV radiation; thus, its waste is nonbiodegradable.

Copper oxide has emerged as a subject of extensive research, primarily due to its versatile properties as a distinct class of crystalline materials^58,59^. The catalytic properties and selectivity of nano-copper oxide surpass those of bulk copper oxide materials. Surface modification of nanoparticles significantly enhances their functionality and effective surface area. Furthermore, these surface alterations are vital for monitoring and controlling particle agglomeration as time progresses. These may provide the nanoparticles with outstanding adsorption capability for dyes and pesticides^60,61^. Copper oxide (CuO) nanoparticles have been extensively researched for numerous years due to their significant functions in catalysis, metallurgy, and high-temperature superconductors.

The present study provides polymeric adsorbents not fabricated before, in addition, based on start cheap materials. The efficacy of these materials towards the uptake of MO is considered acceptable compared with like polymeric materials.

## Experimental

### Materials

The various chemical precursors and reagents required for this research were procured from a range of commercial suppliers. Koch Light Laboratories Ltd. supplied the N-Methyl aniline (98%, d = 0.989 g/mL) and styrene. From Sigma Aldrich (Germany), ammonium persulphate (APS, 98%), 2,2-azobisisobutyronitrile (98%), and dichloromethane (99%) were acquired. Additional chemicals, including, hydrochloric acid (33%), glacial acetic acid, sodium hydroxide (98% pellets), and methanol (99.8%), were provided by the El-Nasr Pharmaceutical Chemical Company in Egypt. N-Methylpyridine (99.5%) was sourced from Oxford Company, India, all solutions and experimental trials were prepared and conducted using distilled water.

### Preparation of the applied samples

#### Preparation of PS

The majority of styrene polymerization was carried out as previously outlined^62^. In summary, 5 g of styrene (5.5mL without inhibitor) and 1 wt % 2,2^\^-azobisisobutyronitrile (0.05 g) were put into a sealed glass tube and secured tightly with a flame. To maintain an inert environment, nitrogen was employed as a purge gas, maintained at a constant flow rate of 50 cm^3^ min^− 1^. The polymerization took place under isothermal conditions at 70 °C for 3 hours. The quantity of unreacted monomer was established by measuring the conversion % (~ 95%) following the processes of dissolving, casting, drying, and weighing the final product. The mean molecular weight (Mw) of the PS was 1,080,000 (g/mol.).

#### Preparation of CuO nanoparticles

Copper oxide nanoparticles (CuO-NPs) were fabricated via chemical precipitation, following a modified established standard procedure^63^. Initially, 9.0 g (0.05 mol) of copper (II) chloride dihydrate and 5.4 g (0.135 mol) of sodium hydroxide pellets were dissolved independently in a minimum volume of ethanol. The sodium hydroxide solution was introduced dropwise into the copper (II)chloride solution under constant magnetic stirring at room temperature. During this process, a distinct color shift was observed, transitioning from green to bluish-green and finally to a black precipitate, signifying the formation of copper hydroxide. The isolation of the solid product was achieved through centrifugal separation, after which the samples were rinsed extensively with deionized water and ethanol to ensure the removal of any byproduct salts. After drying the specimen at 50 °C, the resulting powder was annealed across a temperature gradient of 200 °C, 400 °C, and 600 °C. Finally, manual milling was performed to refine the crystalline CuO nanoparticles.

#### Preparation of PS/PNMANI/CuO composite

The PS solution (4 g PS dissolved in 100 mL dichloromethane) was stirred at 25 °C and with 500 rpm, and then 0.4 g CuO powder was added to the suspension in the PS solution. 4.0 g (4.0 mL) of N-methylaniline was added to the above PS solution, and after the complete disappearance of N-methylaniline, 2 mL glacial acetic acid was added. Finally, 8.0 g APS was added^41^. Chemical oxidative polymerization of N-methylaniline in the presence of both PS and CuO NPs was performed at room temperature. The reaction was kept constant with stirring for 3 h, and then left overnight. A dark violet precipitate of the composite was formed, and isolated by filtration, rinsed thoroughly with distilled water and methanol and followed by thermal drying in an oven at 100 °C for 24 h.

#### Preparation of PS/PNMANI blend

The PS/PNMANI blend was synthesized as described above Sect. 2.2.3 without addition of CuO NPs.

#### Stock solution preparation

A standard stock solution of Methyl Orange (MO) at a concentration of 40 ppm was prepared by dissolving 0.0400 g of the dye in 1000 mL of distilled water. Subsequently, this primary solution was utilized to create a series of working solutions at the required initial concentrations through a systematic dilution process.

### Instrumentation

The structural properties of the prepared PS/PNMANI and PS/PNMANI/CuO composites were investigated using X-ray diffraction (XRD) with an APD-3720 diffractometer operating with Cu Kα radiation. The measurements were carried out at 40 kV and 20 mA, scanning a 2θ range from 10° to 70° at a scanning rate of 2° per minute.

Surface morphology was examined using scanning electron microscopy (SEM, JSM 6700 F, JEOL), while the functional groups and molecular vibrations were identified through FTIR analysis, FTIR spectra were given at room temperature over in range between 400 and 4000 cm^−^¹ using the Shimadzu FTIR Vertex70 Bruker optical technique. In addition, nitrogen adsorption–desorption isotherms was used to determine the specific surface area was studied using a TriStar II 3020 analyzer at 77 K.

Thermogravimetric analysis (TGA) was performed using a Shimadzu TGA-50 H instrument equipped with a platinum sample holder at a heating rate of 20 °C/min under nitrogen atmosphere to assess the thermal decomposition behavior.

### Batch adsorption experiments

To establish the optimal parameters for MO adsorption using the studied materials, several experimental variables were investigated as summarized in Table 1. These included the starting MO concentration, adsorbent dosage, stirring time and the initial pH value of the solution (ranging from 3 to 10). Post-equilibrium, the residual MO concentration was quantified by measuring the absorbance of the supernatant at 460 nm using a DR 6000 dual-beam UV-visible spectrophotometer. The removal efficiency (R%) and the amount of dye adsorbed at equilibrium and qe (mg/g) were subsequently determined using Eqs. (1) and (2).

1 $$\:\mathrm{R\:\%\:=\:}\frac{\mathrm{\:(C}_\mathrm{i}\text{}-\mathrm{\:C}_\mathrm{e}\mathrm{)}}{\mathrm{C}_\mathrm{i}}\:\times\:100$$

2 $$\:\mathrm{q}_\mathrm{e}\mathrm{\:=\:}\frac{\mathrm{V(C}^\circ_\mathrm{i}\mathrm{\:\:-\:C}_\mathrm{e}\mathrm{)}}{\mathrm{m}}$$

As Ce (mg/L) represents the equilibrium dye concentration, while Ci denotes the initial MO concentration in water. V = volume of solution, and m = mass of the adsorbent used.

**Table 1:** Summarizes the experimental conditions applied during the study, along with the corresponding experimental conditions used.

studied parameter	Conditions						Other parameters
pH	2	5	7	10	12		initial conc. of MO = 10 mg/L, dose ranged from 50 to 150 mg, contact time/speed is 20 min/300 rpm, total volume = 100 mL
Dose (mg)	20	ranged		200	250		100 mL volume of pH = 7.0, 10 mg/L of MO initial concentration, and contact time = 60 min with speed of 300 rpm
Agitation time (min.)	5 10	15	20	30	60		pH (7.0), 50–150 mg dosage, 100 mL total volume, 300 rpm agitation speed, starting concentration of 10 mg/L of MO
MO initial conc., (mg/L)	2.5	5	7.5	10	12.5		pH (7.0), 2.5–12.5 mg dose, 60 min of agitation = 300 rpm, and 100 mL of total volume

### Adsorption studies

Batch adsorption tests were conducted for evaluate the efficiency of PS/PNMANI and PS/PNMANI/CuO composites under various experimental conditions. A predetermined amount of the polymeric adsorbent was added to 100 mL Erlenmeyer flasks containing MO solutions, and the optimum operating parameters, including pH, adsorbent dose, and initial dye concentration, were systematically determined. A 40 ppm MO stock solution was prepared, and the required working concentrations were obtained through serial dilution with distilled water. The remaining concentration of MO in solution was detected by DR 6000, dual-beam UV–visible spectrophotometer after system reached equilibrium at a wavelength of 460 nm. The effects of key parameters such as initial dye concentration, adsorbent dosage, contact time, temperature, and solution pH on the adsorption performance were thoroughly investigated. The removal efficiency was calculated using Eq. 1. For the kinetic study, the adsorption behavior of MO was analyzed using pseudo-first-order (PFO) and pseudo-second-order (PSO) models. Adsorption isotherms, which are essential for understanding the adsorption mechanism and affinity toward MO^41^, were evaluated using ‘Langmuir, Freundlich, and Temkin models.

## Results and discussion

### Characterization

**Fig. 1:**
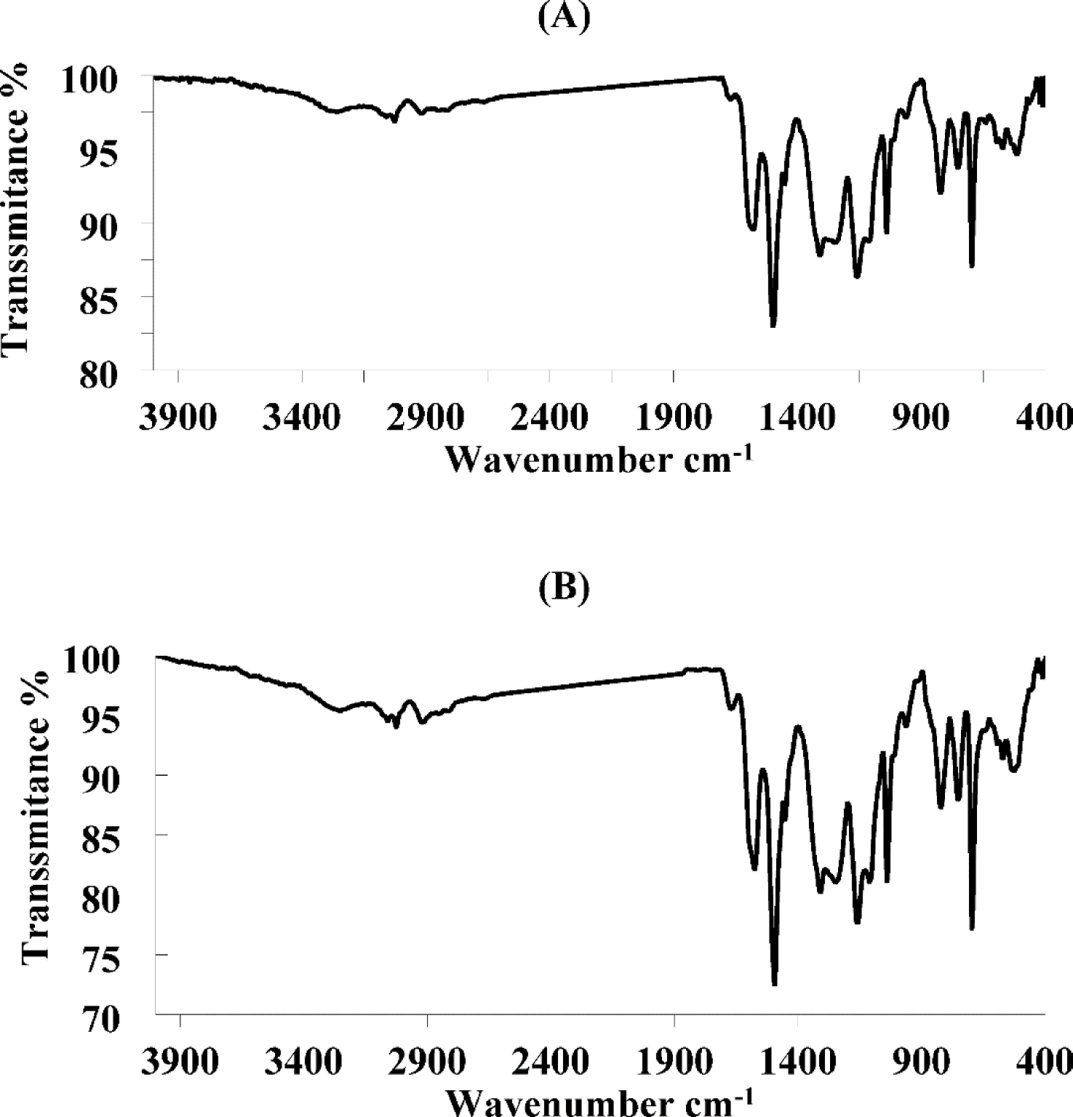
IR spectra of **(A)** PS/PNMANI and **(B)** PS/PNMANI/CuO.

Figure 1 presents the FTIR spectra of PS/PNMANI and PS/PNMANI/CuO. The absorption bands observed at 1494 and 1574 cm⁻¹ correspond to the C = C and C = N stretching vibrations of the benzenoid and quinoid structures in PS and/or PNMANI, respectively, showing clear shifts in both position and intensity compared to pure PNMANI^64–67^. The band at 1295 cm^− 1^ is assigned to C–H stretching of the benzenoid ring. Vibrations in the 1100–1400 cm^− 1^ region are linked to the –NCH_3_ functional group. A weak characteristic peak at 3250 cm^− 1^ is associated with C–H stretching vibrations of the aromatic ring, while additional bands at 2918 cm^− 1^ and 3025 cm^− 1^ are related to symmetric and asymmetric stretching and out-of-plane bending of CH_3_ and aromatic C–H groups. The C–H stretching of aliphatic CH_3_ groups appears in the range of 1450–1600 cm^− 1^. Moreover, C = C stretching vibrations of the aromatic ring (typically observed at 1601, 1494, and 1451 cm^− 1^) exhibit noticeable shifts in both position and intensity when compared to pure polystyrene. The band at 571 cm^− 1^ in the nanocomposite shows a red shift relative to pure CuO nanoparticles, confirming Cu–O stretching vibrations. Overall, the observed changes in the characteristic peaks of PS/PNMANI and PS/PNMANI/CuO indicate strong interactions between the components of the composite.

**Fig. 2:**
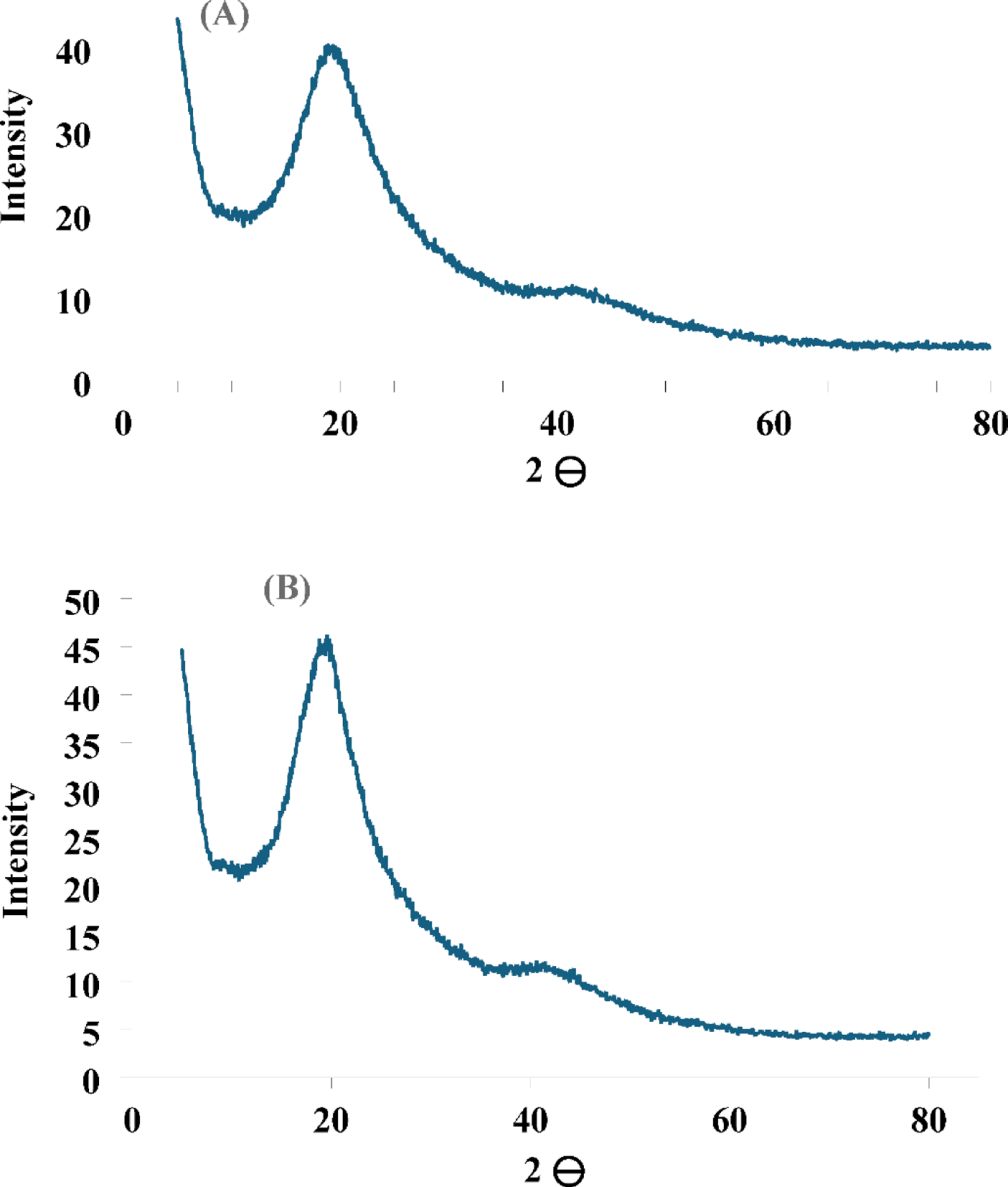
XRD patterns of **(A)** PS/PNMANI and **(B)** PS/PNMANI/CuO.

Figure 2 displays the XRD pattern for PS/PNMANI and PS/PNMANI/CuO. The XRD diffractogram of PS/PNMANI shows a wide peak centered at 20º, as illustrated in the Fig. 2(a). These peaks correspond to the (020) plane of PS/PNMANI aligned with JCPDS card No 53–1891^68^, indicating that PS/PNMANI possesses a semi-crystalline structure^69^, which highlights the absence of an additional peak when compared to pure PNMANI. The PS spectrum exhibits a semi-crystalline characteristic with a sharp peak at 10.19°, and the loss of this peak indicates a chemical interaction between PS and PNMANI. Many of the CuO structures exhibit a range of peaks over 2⊖=30(38.37, 35.25) with varying intensities. The vanishing of the CuO peaks in the PS/PNMANI/CuO composite signifies that it is integrated, enveloped, and distributed within the structure of PS/PNMANI/CuO^70^(Fig. 2B). Tables 2 and 3 illustrate the crystallite sizes of PS/PNMANI and the PS/PNMANI/CuO composite respectively.

**Table 2:** Crystallite size of PS/PNMANI.

2⊖	B in degree	B in radians	D nm
60.7443	0.456	0.0079	20.3
18.205	0.1	0.0017	82.56
19.133	0.1	0.0017	82.67
20.472	0.1	0.0017	82.8
21.163	0.1	0.0017	82.9
24.940	0.1	0.0017	83.4

**Table 3:** Crystallite size of PS/PNMANI/CuO.

2⊖	B in degree	B in radians	D nm
15.994	0.1	0.0017	82
18.728	0.1	0.0017	82.6
19.489	0.1	0.0017	82.7
20.021	0.1	0.0017	82.78

**Fig. 3:**
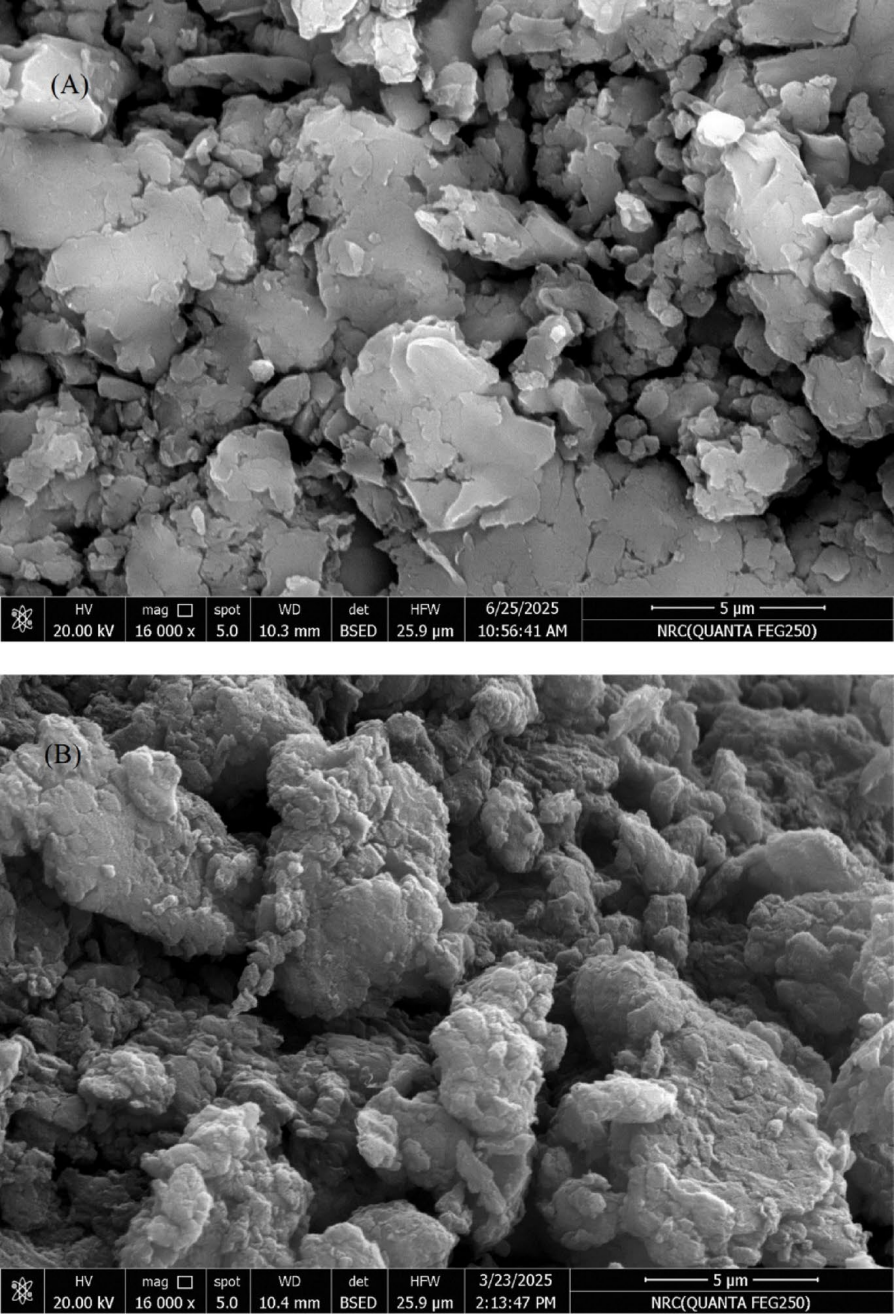
SEM pictures of **(A)** PS/PNMANI and **(B)** PS/PNMANI/CuO.

**Fig. 4:**
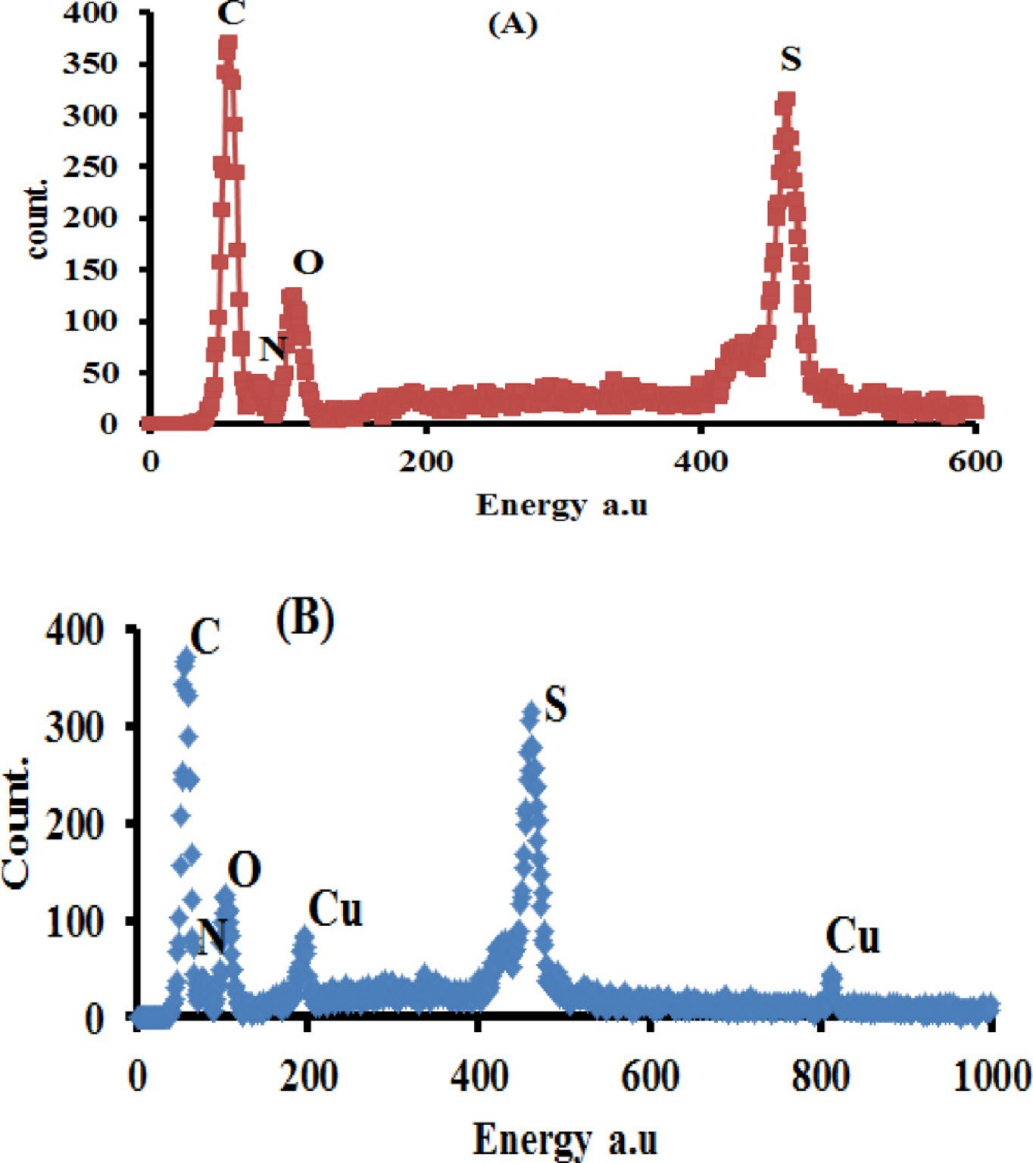
EDAX data of PS/NMANI **(A)** and PS/NMANI/CuO **(B)**.

Figure 3, A and B present scanning electron microscopic images of PS/PNMANI and PS/PNMANI/CuO respectively. Image A indicates that PS/PNMANI is primarily made up of dense sheets along with various spherical grains of different dimensions, and the inclusion of PNMANI in the composition results in uniform surfaces exhibiting varying pore sizes. Image B of PS/PNMANI/CuO displays various morphologies influenced by the presence of CuO within the copolymer segment, with grains resembling each other in shape and size. The particles remain dense, and the pores differ from those illustrated in Figure A. The EDAX analysis indicates the existence of Cu in sample PS/PNMANI/CuO, confirming the composite’s formation (refer to Figs. 4A and B, and Table 4). The particle size of PS/PNMANI/CuO ranged from 2.32 to 3.56 microns and for PS/PNMANI ranged from 2.5to 3.75 microns.

**Table 4:** Elemental composition of investigated samples from EDAX.

Sample	Element weight%				
	C	*N*	O	S	Cu
**Sample A**	48.38	20.61	22.78	8.22	-
**Sample B**	47.96	27.83	18.27	5.52	0.41

**Fig. 5:**
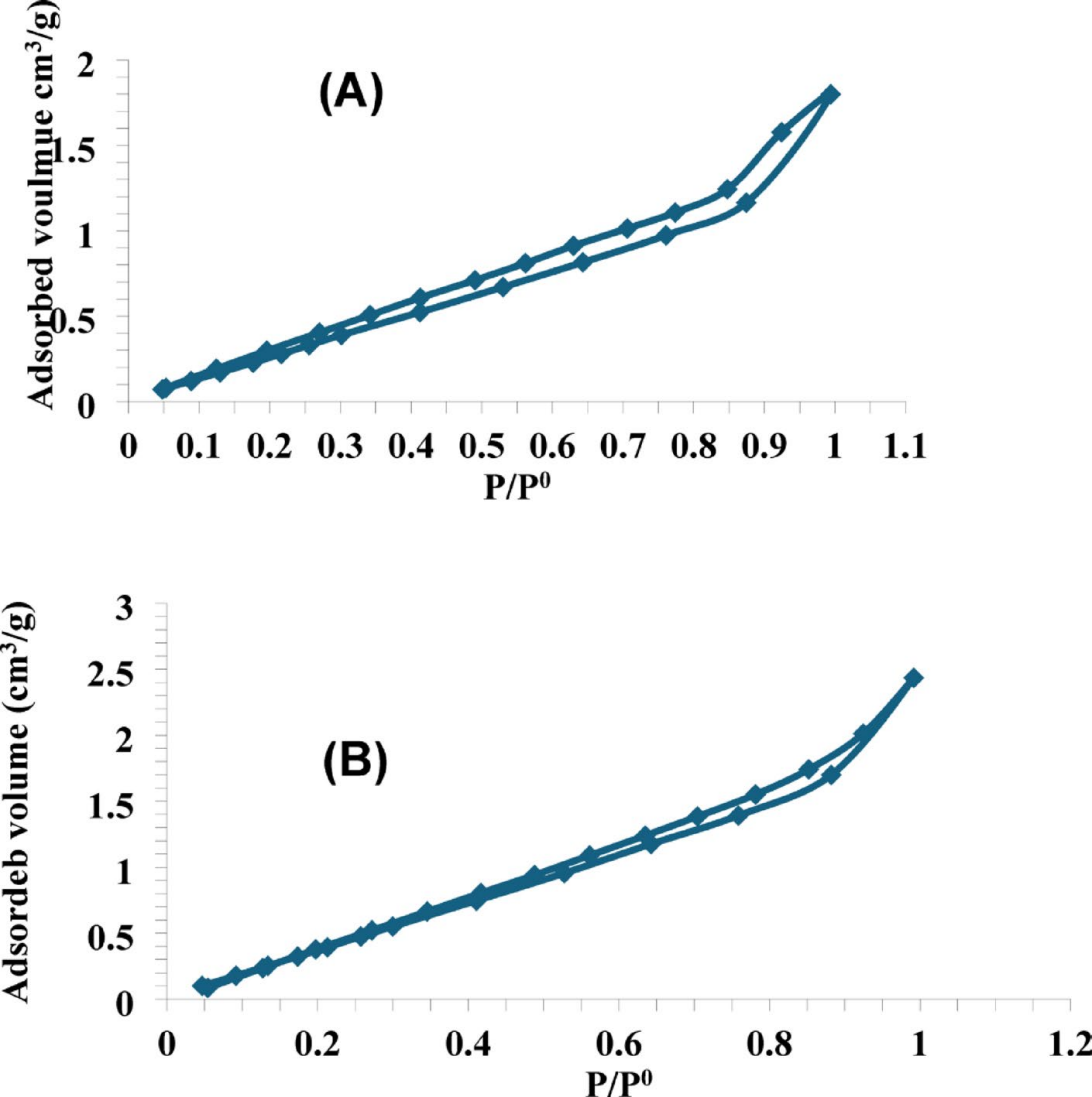
Adsorption-desorption behaviour of PS/PANMANI **(A)** and PS/PNMANI/CuO **(B)**.

A gas sorption instrument, TriStar II 3020 from Micrometrics, was employed to assess textural properties via the BET method for the PS/PNMANI and PS/NMANI/CuO composites. N_2_ adsorption–desorption isotherms had been carried out to evaluate specific surface area and total pore volume as presented in Fig. 5A & B. According to IUPAC classification, the obtained N_2_ adsorption–desorption curves correspond to a type IV isotherm accompanied by an H_3_ hysteresis loop^71^. The Type IV isotherm signifies the mesoporous characteristics of the synthesized materials. Table 5 indicates that the surface area rose with the addition of CuO into the PS/PNMANI structure, alongside increases in total pore volume, which can be linked to the improvement of the surface and reinforcing the composite strong adsorption capacity towards MO.

**Table 5:** BET data of the synthesized materials.

BET parameter	Investigated sample	
	PS/PNMANI	PS/PNMANI/CuO
Surface area (m^2^/g)	34	42
Total pore volume (cm^3^/g) at p/p^0^ = 0.95.	0.036	0.040

**Fig. 6:**
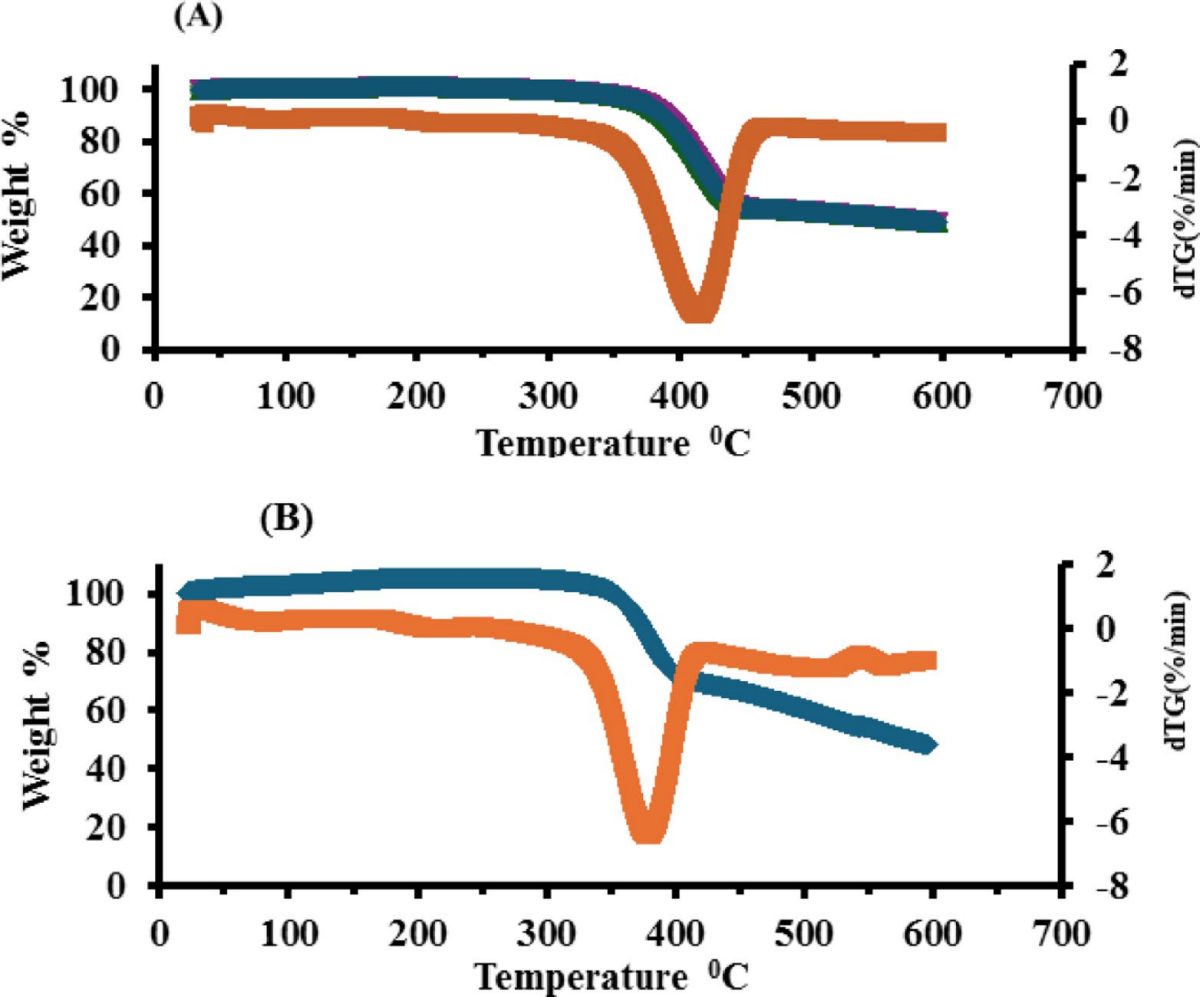
TGA for PS/PNMANI **(A)** and PS/PNMANI/CuO **(B)**.

Thermogravimetric analyses (TGA) for both PS/PNMANI and PS/PNMANI/CuO are shown in Fig. 6A&B. The thermal stability results indicate that the synthesized polymeric samples maintained thermal stability up to ~ 350 °C with minimal weight loss. Above 350 °C, the current samples experience degradation with a significant weight loss of 40%, with a midpoint at 420 °C for PS/PNMANI, and approximately 25%, with a midpoint at 380 °C for PS/PNMANI/CuO. The degradation’s annealing phase for PS/PNMANI/CuO commenced at 400 °C, resulting in a cumulative mass loss 50%. The residual weight% at 600 °C was 60 for PS/PNMANI and 50 for PS/PNMANI/CuO. The thermal properties of the two samples suggested the inclusion of CuO in the PS/PNMANI sample and the formation of carbon residue.

### Adsorption studies

Each run was performed three times and the mean was used for study.

#### Effect of contact time

**Fig. 7:**
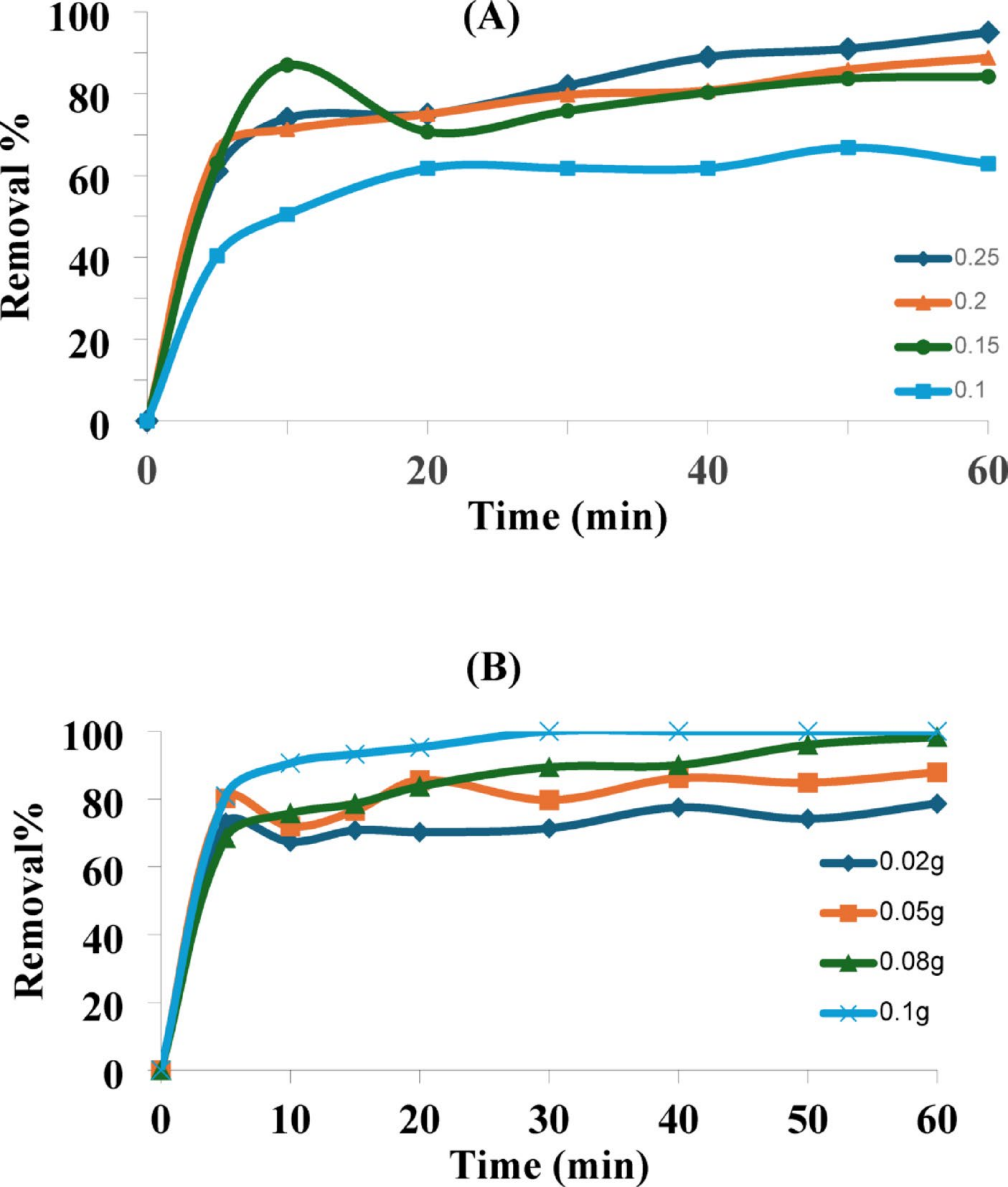
Effect of contact time on the removal of MO at different doses of **(A)** PS/PNMANI and **(B)** PS/PNMANI/CuO.

**Fig. 8:**
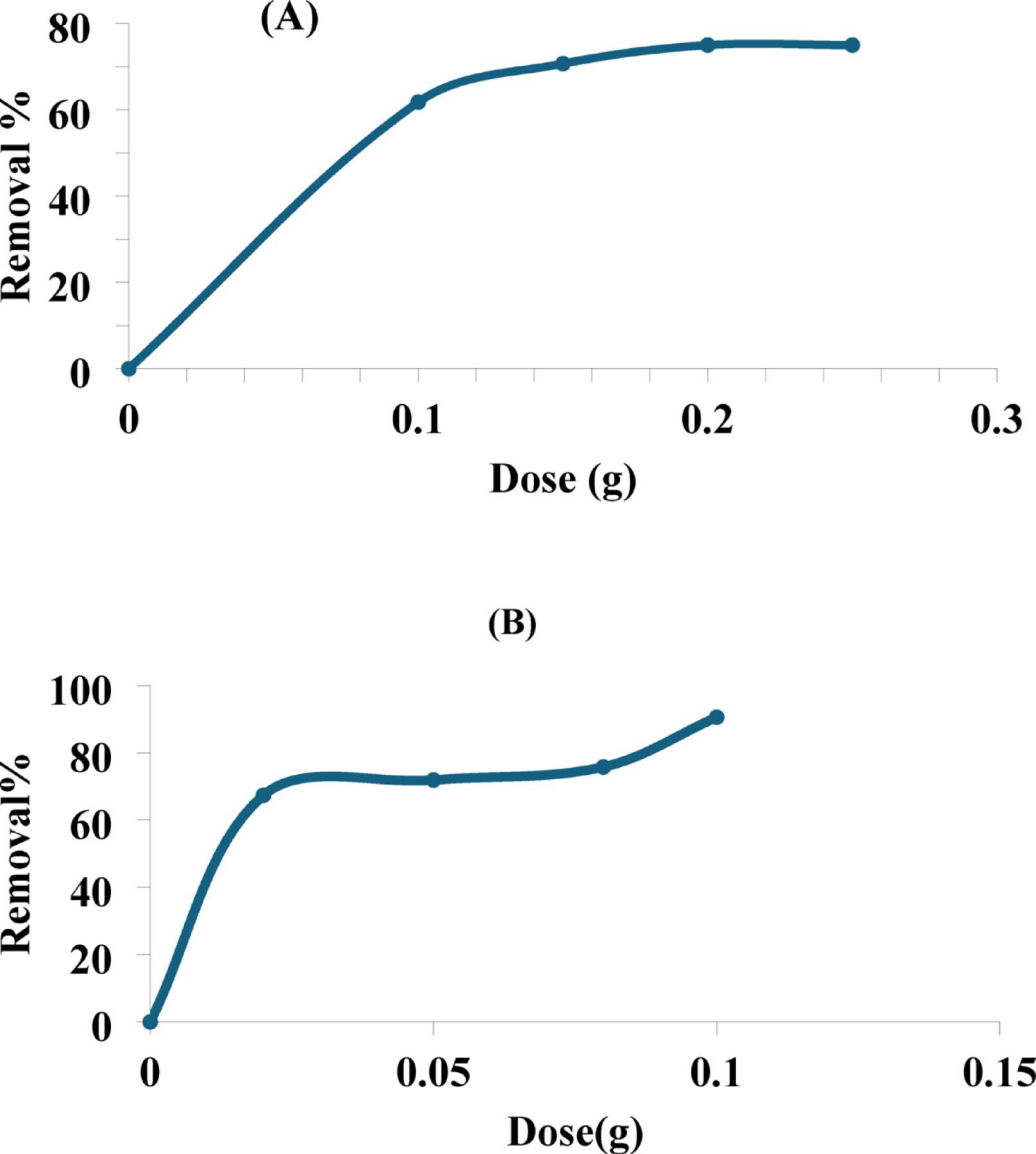
Effect of dose on the removal of MO **(A)** PS/PNMANI and **(B)** PS/PNMANI/CuO.

The effectiveness of PS/PNMANI and PS/PNMANI/CuO in eliminating MO was examined over various time intervals, and the obtained results are depicted in Fig. 7. The removal was conducted at ambient temperature and pH = 7 with varying amounts of polymeric samples. The MO concentration was modified to 10 ppm while examining factors in the removal process. The amounts of PS/PNMANI are 0.10, 0.15, 0.20, and 0.25 g/100 mL MO solution; for PS/PNMANI/CuO, the amounts are 0.02, 0.05, 0.08, and 0.1 g/100 mL MO solution. The duration of contact was 60 min. The percentage of removal was computed and is visually displayed in Fig. 7. The collected data indicate that the majority of the MO is eliminated within the initial contact period, as the sorption process quickly progresses to full covering the active sites. Furthermore, by raising the sample dose, the removal percentage rose to 80% within 10 min, as indicated in Fig. 8. This can be justified by enhancing the sorbent surface area and quantity. After 10 min, the removal percentage stays the same, which is due to the full occupation of the adsorbent surface sites. For PS/PNMANI, 0.25 g can eliminate approximately 90% of the dye; however, for its composite with CuO, the amount required was nearly 0.10 g. Moreover, the influence of the adsorbent amount was assessed at pH = 7 and ambient temperature.

#### Effect of pH

**Fig. 9:**
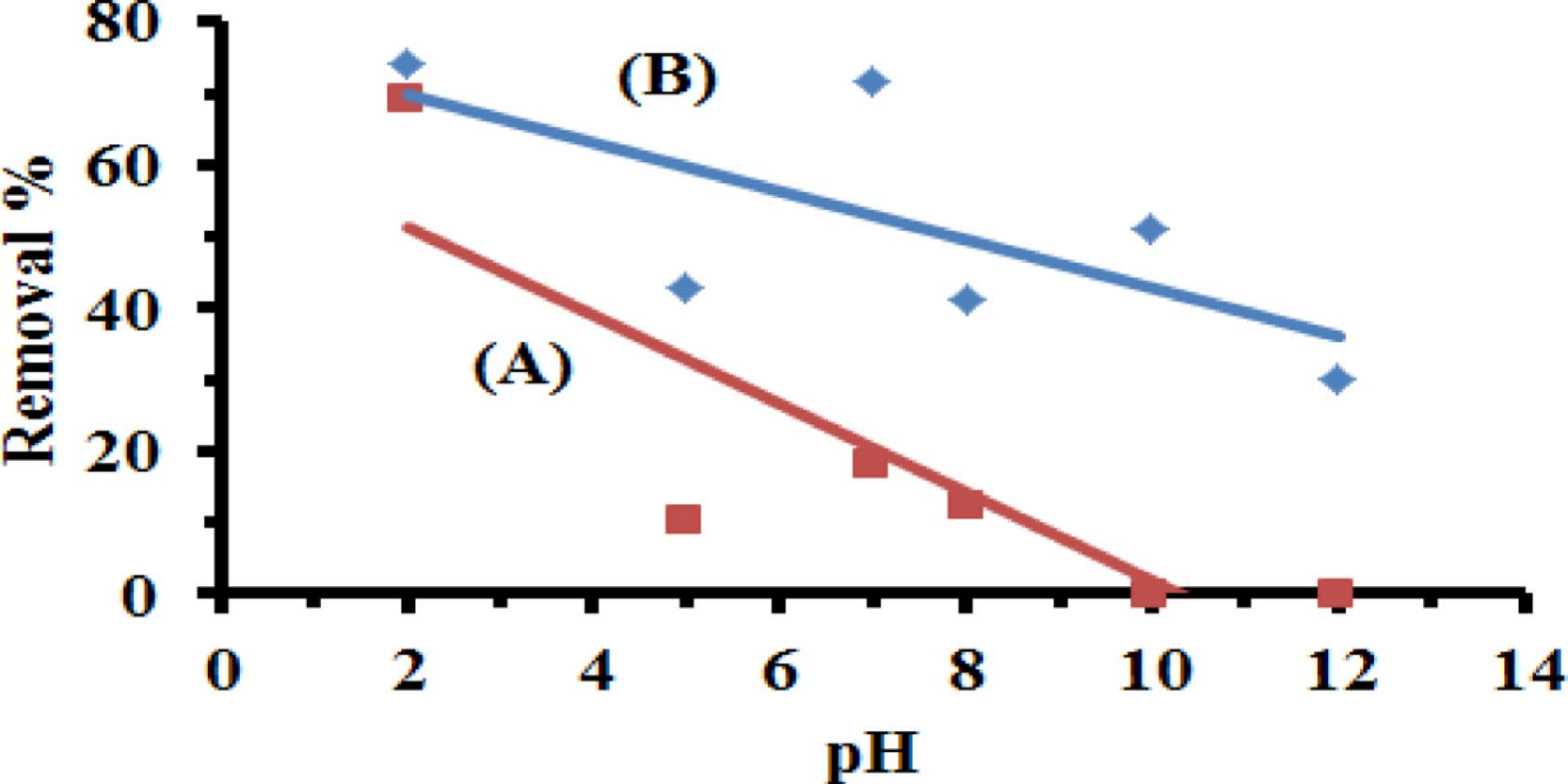
Effect of pH on removal efficacy of PS/PNMANI **(A)** and PS/PNMANI/CuO **(B)**.

**Fig. 10:**
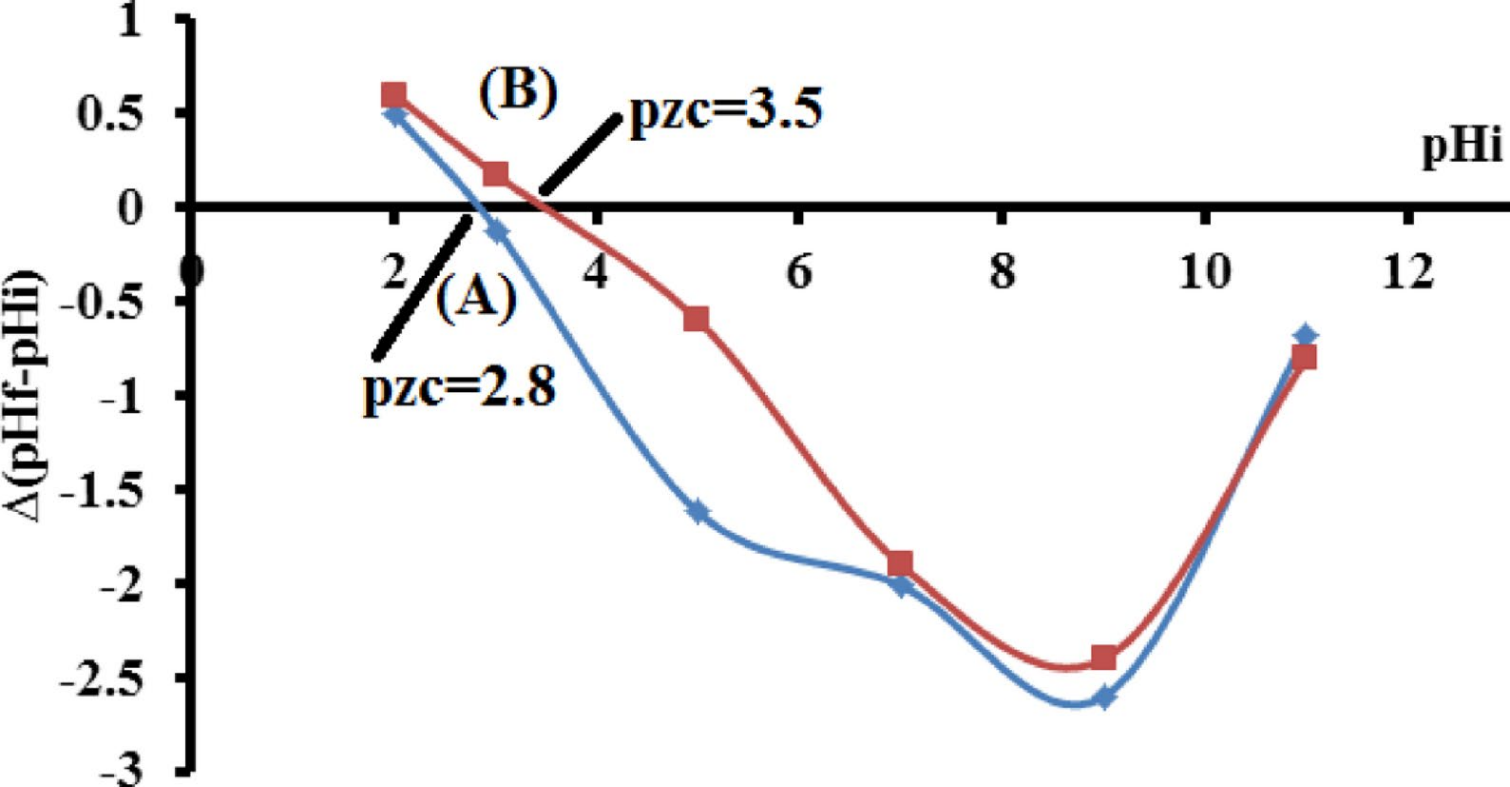
PH _pzc_ of PS/PNMANI **(A)** and PS/PNMANI/CuO **(B)**.

Figure 9 illustrates the influence of the pH of the MO solution on the adsorption performance of both PS/PNMANI and PS/PNMANI/CuO. An earlier study demonstrated that pH exhibited a variable impact on MO removal. Overall, the removal percentage declines as pH rises, which can be linked to the alteration in the MO moiety structure resulting from its ionization level. The selected adsorbents’ charged active sites interact electrostatically with the MO due to these conditions. The adsorption behavior of MO, an anionic dye, is fundamentally governed by the surface charge of the composites, which is characterized by the point of zero charge (pHpzc). As illustrated in Fig. 10, the pHpzc values for PS/PNMANI and PS/PNMANI/CuO were identified at 2.8 and 3.5, respectively. At pH levels below these values, the protonation of functional groups imparts a positive charge to the adsorbent surfaces, significantly enhancing the electrostatic attraction toward the negatively charged MO molecules. Conversely, increasing the solution pH leads to a gradual reduction in positive sites and an increase in negative surface charge, resulting in electrostatic repulsion and a marked decline in adsorption capacity. This confirms that acidic environments (pH < pHpzc) are optimal for MO uptake, where electrostatic interactions serve as the predominant mechanism.

#### Effect of temperature and thermodynamics

**Fig. 11:**
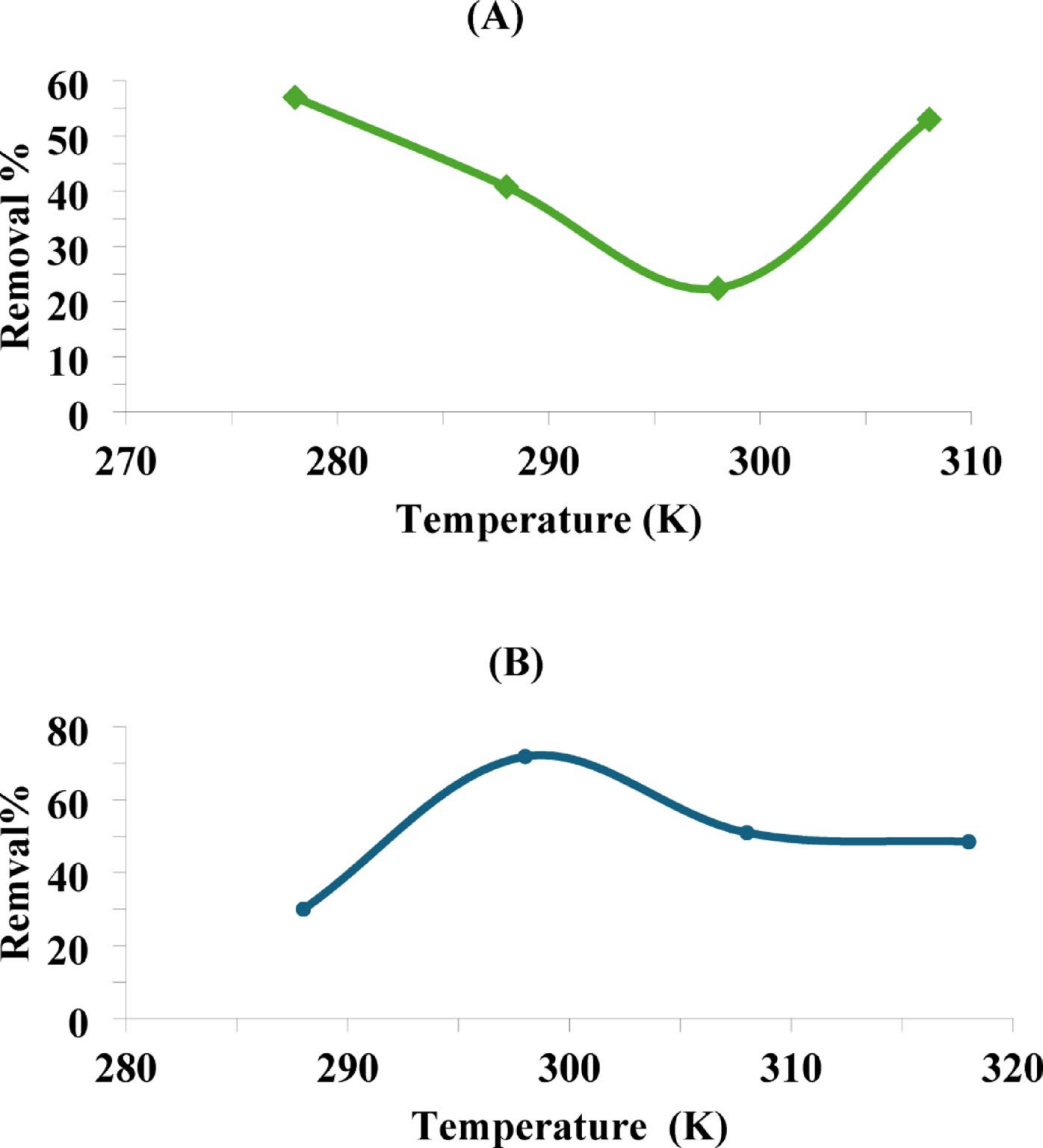
Effect of temperature of the removal of MO using PS/PNMANI **(A)** and PS/PNMANI/CuO **(B)**.

The influence of temperature between 15 and 45 °C on MO (10 ppm) absorption from a water source using 0.15 g PS/PNMANI and 0.05 g of PS/PNMANI/CuO was investigated at pH = 7 for 10 and 20 min with 10 ppm MO, respectively. The information collected at various temperatures is illustrated in Fig. 11. The findings reveal that the removal % reduced from 5 °C to 25 °C and then rose to 35 °C for PS/PNMANI, while it rose from 5 °C to 25 °C and then fell for PS/PNMANI/CuO. Typically, the chemical adsorption process is exothermic, and a rise in temperature opposes the process; the differences observed in this study may be due to the combined types of adsorption and various removal mechanisms. The thermodynamic variables were derived from this equation^69^.

3 $$\:\varDelta\:\mathrm{G}\circ\:=\:-\:\mathrm{R}\mathrm{T}\:\mathrm{L}\mathrm{n}\:\mathrm{K}\mathrm{c}$$

4 $$\:\varDelta\:\mathrm{G}\circ\:=\:\varDelta\:\mathrm{H}^0\hspace{0.17em}-\hspace{0.17em}\mathrm{T}\varDelta\:\mathrm{S}^0\:$$

5 $$\:\mathrm{L}\mathrm{n}\:\mathrm{K}\mathrm{c}\:=\frac{\varDelta\:\mathbf{S}0}{\boldsymbol{R}}+\frac{\varDelta\:\mathbf{H}0}{\boldsymbol{R}\boldsymbol{T}}$$

**Fig. 12:**
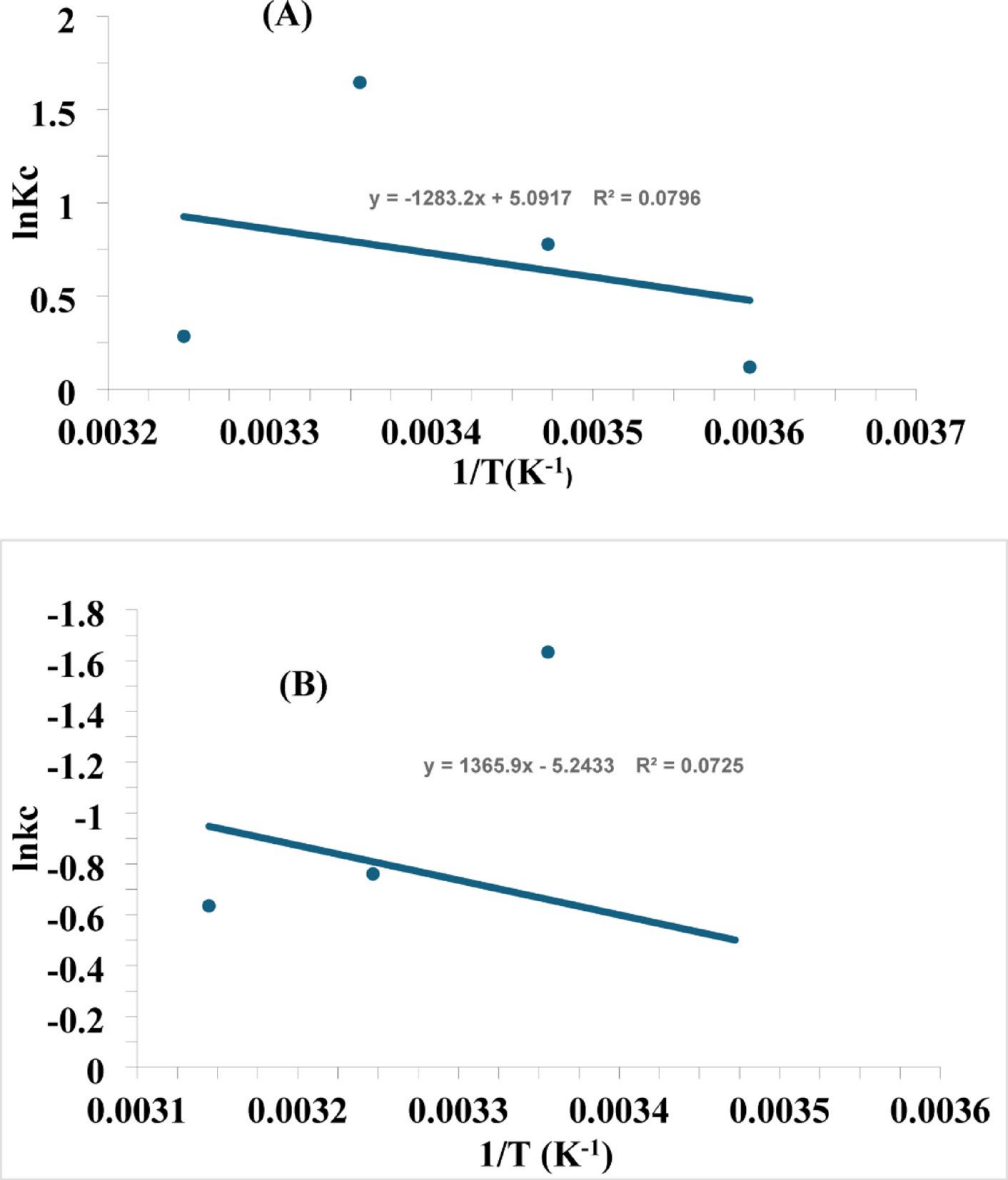
Vant Hoff plots for the adsorption of MO on PS/PNMANI **(A)** and PS/PNMANI/CuO **(B)**.

As R represents the universal gas constant (8.314 J mol^–1^ K^–1^), T represents the absolute temperature in Kelvin, Kc represented Langmuir constant, while ΔH^0^ and ΔS^0^ correspond to standard enthalpy and entropy changes of the adsorption process, respectively. The thermodynamic parameters ΔH^0^ and ΔS^0^ were determined from the linear relationship between ln Kc and 1/T, as illustrated in Fig. 12^72^. The obtained values are summarized in Table 6.

The ΔH^0^ values reported in Table 6 indicate that the adsorption of MO onto PS/PNMANI/CuO is exothermic, whereas it is endothermic in the case of the copolymer PS/PNMANI which can be attributed to consumption of energy production from the bonding between MO groups (sites) and PS/PNMANI sites to free further sites from bonded water. From Temkin BT constant (Table 7) the value compared with composite with CuO is very low which confirm the lack of energy during the adsorption of MO as monolayer on PS/PNMANI so the energy of surrounding is decrease. In addition, the negative Gibbs free energy values verify that the adsorption process proceeds spontaneously.

**Table 6:** Thermodynamic parameters of MO adsorption on the investigated polymeric samples.

Parameters		Polymeric sample	
		PS/PNMANI	PS/PNMANI/CuO
Temperature(K)	degree	∆G (J/mole)	
	288	−1.5232	−1.1986
	298	−1.9466	−1.6345
	308	−2.3699	−2.0704
	318	−2.7932	−2.5063
∆H(kJ/mole)		10.6685	−11.3561
∆S (J. mole^− 1^.K^− 1^)		42.3324	−43.5927

#### Effect of MO initial concentration on adsorption capacity

**Fig. 13:**
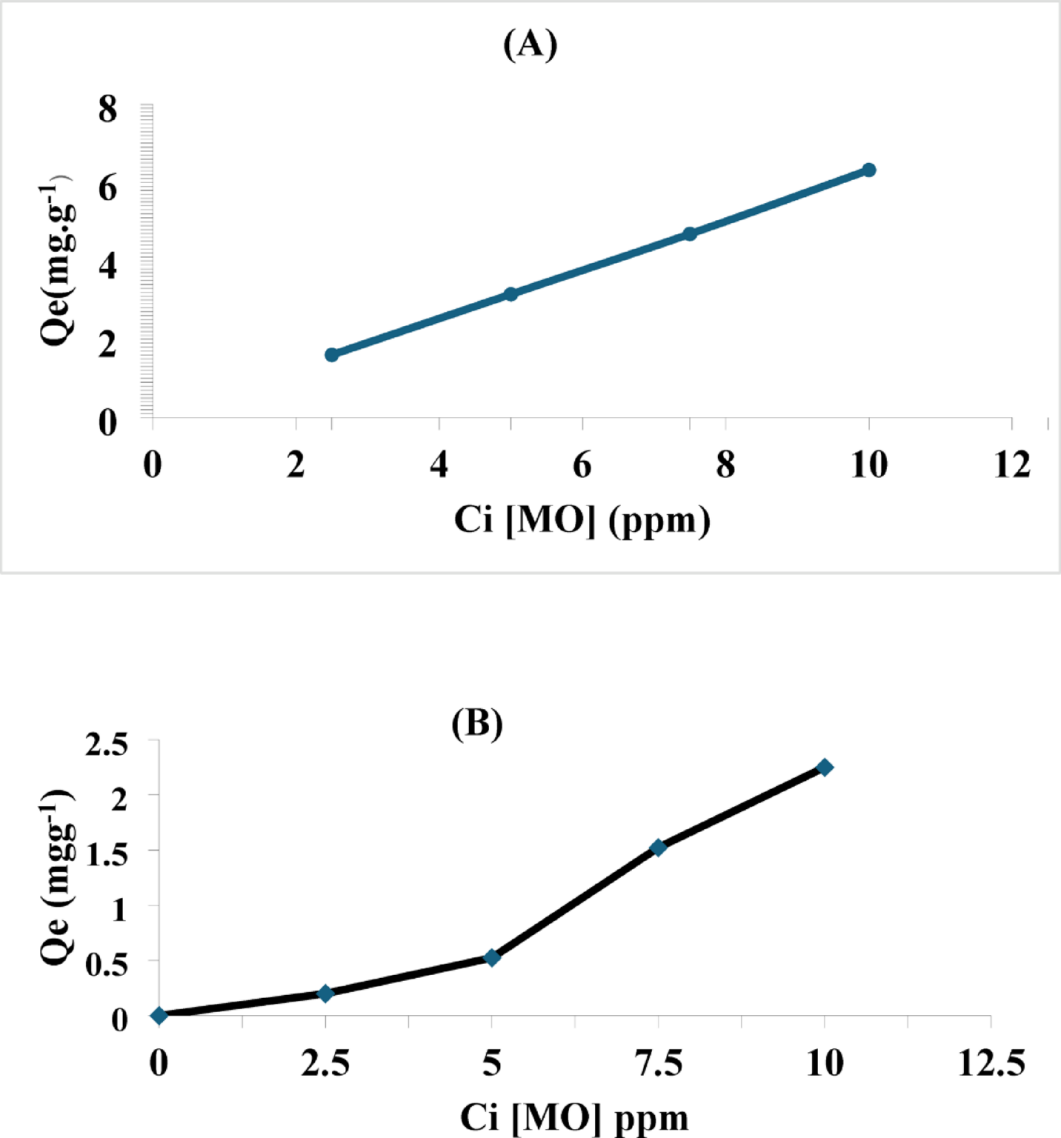
Effect of MO initial concentration on adsorption capacity of PS/PNMANI **(A)** and PS/PNMANI/CuO **(B)**.

The effect of **initial** MO concentration on adsorption ability of the synthesized polymeric samples was examined. The starting MO concentration ranged from 2.5 to 15 ppm, and the findings are illustrated in Fig. 13. A direct relationship was identified between the starting dye levels and the quantity adsorbed, with the system exhibiting enhanced uptake as concentrations rose from 2.5 to 10 ppm. This rise was warranted by a rise in the quantity of adsorbate molecules surrounding the adsorbent, which aided in the attraction of the active sites at the adsorbent/adsorbate interface^73,74^. As the initial concentrations increase, the absorption capacity also increases, while the adsorption percentage decreases because of surface saturation^75^.

#### Adsorption kinetics

Adsorption investigations of MO on PS/PNMANI and PS/PNMANI/CuO are conducted to assess the adsorption exponent. In this study, adsorption kinetics were characterized via two commonly applied models. The first is the pseudo-first-order model^76^, which relates the adsorption rate to the number of available active sites, accounting for the variation in unoccupied sites over time (Eq. 6). The second is the pseudo-second-order model^77,78^, which describes how the adsorption capacity evolves as a function of time (Eq. 7).

6 $$\:\mathrm{L}\mathrm{n}\:(\mathrm{q}\mathrm{e}\hspace{0.17em}-\hspace{0.17em}\mathrm{q}\mathrm{t})\hspace{0.17em}=\hspace{0.17em}\mathrm{L}\mathrm{n}\:\mathrm{q}\mathrm{e}\hspace{0.17em}-\hspace{0.17em}\mathrm{K}1\mathrm{t}\:$$

7 $$\:\frac{\mathrm{t}}{\mathrm{q}\mathrm{t}}=\frac{1}{\mathrm{k}2\mathrm{q}\mathrm{e}}+\frac{\mathrm{t}}{\mathrm{q}\mathrm{e}}$$

In this context, *k2* (min^− 1^) corresponds to the rate constant of the pseudo-first-order model, while *qe* and *qt* (mg g^− 1^) represent the amounts of metal ions adsorbed at equilibrium and at a given time *t* (min), respectively. The parameter *k2* (g mg^− 1^ min^− 1^) refers to the rate constant associated with the pseudo-second-order model^78^.

**Fig. 14:**
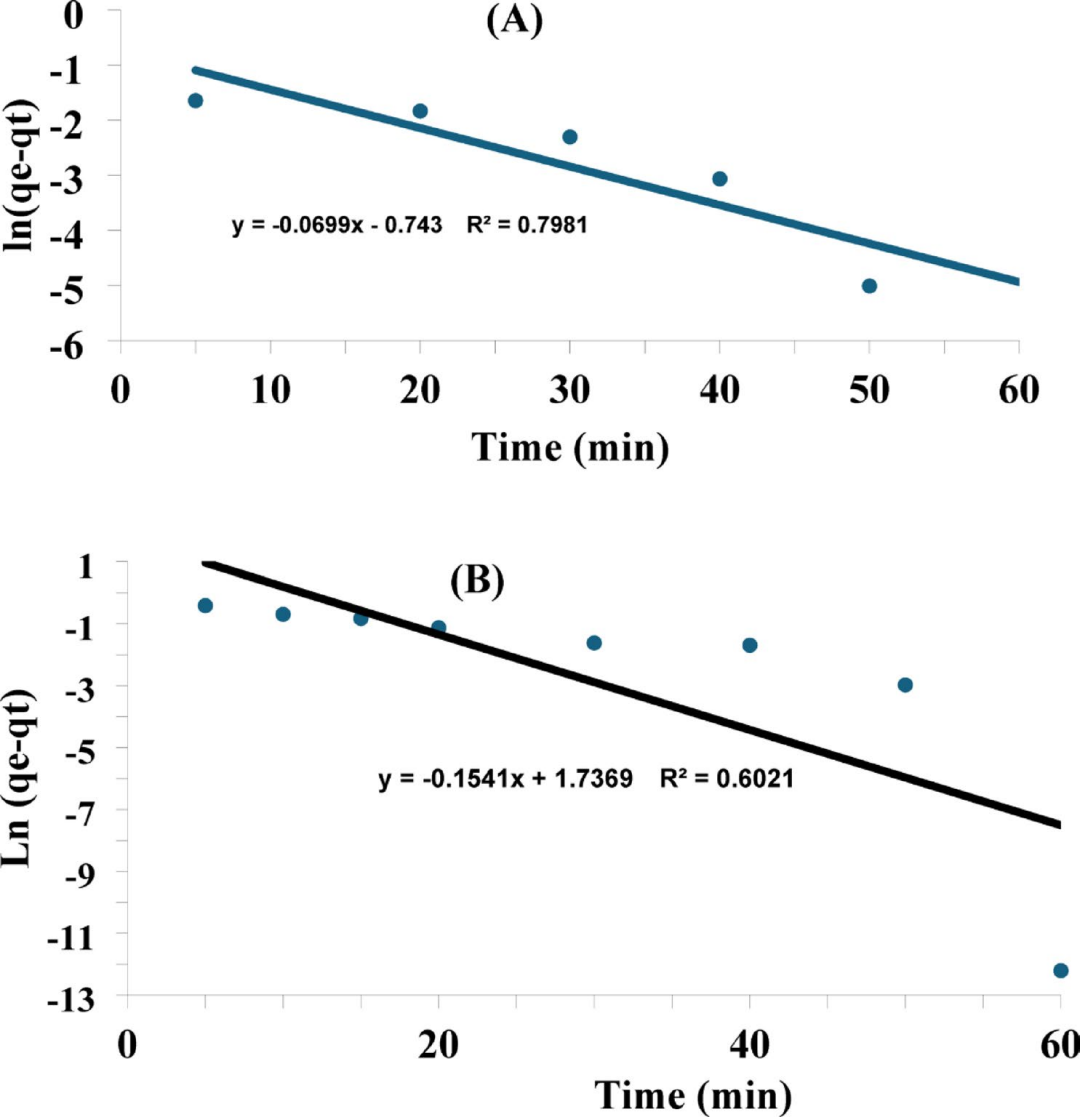
Pseudo-first order model for PS/PNMANI **(A)** and PS/PNMANI/CuO **(B)**.

**Fig. 15:**
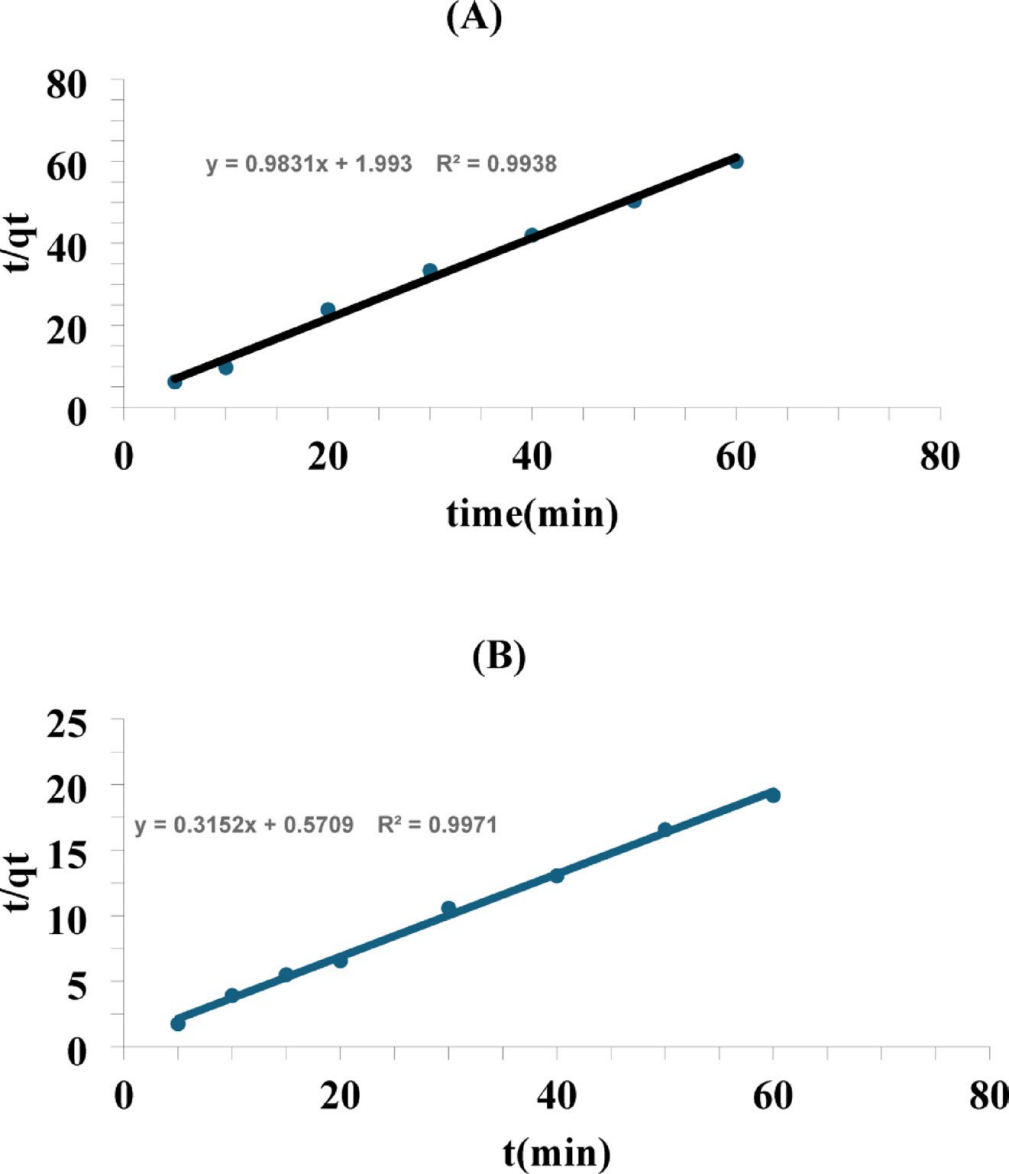
Pseudo-second order model for PS/PNMANI **(A)** and PS/PNMANI/CuO **(B)**.

Figures 14 and 15 illustrate the linear forms of both kinetic models. The corresponding parameters for the first and second order models were derived from the slopes and intercepts of the linear plots of ln(qₑ − qt) versus *t* and (*t/qt*) versus *t*, with the calculated values summarized in Table 8.

From the data presented in Figs. 14 and 15, along with the correlation coefficient (R²), it can be concluded that the adsorption of MO onto the PNMA/PS/CuO surface follows the Lagergren pseudo-second-order kinetic model. This suggests that the removal mechanism involves a combination of chemisorption and physisorption processes. Such behavior is attributed to the presence of lone pair and π-electrons within the polymer structure, particularly at NH, NH_2_, and aromatic (benzene or quinoid) groups. Additionally, the adsorption process may also involve hydrogen bonding and/or electrostatic interactions.

**Table 7:** Isothermal parameters for the adsorption of MO.

Adsorption model	Isothermal parameter	Polymeric samples	
		PS/PNMANI	PS/PNMANI/CuO
Langmuir	Q_m_(mg.g^− 1^)	7.17	43.29
	b	1.91	0.683
	R^2^	0.9928	0.8834
	ARE%	7.8	22.50
	SSE	1.94	156.30
	X^2^	0.42	8.94
Freundlich	n	3.80	1.89
	K_f_(mg.g^− 1^)	2.20 × 10^4^	3860
	R^2^	0.9257	0.9429
	ARE%	28.3	8.20
	SSE	19.8	29.50
	X^2^	5.21	1.15
Temkin	B_T_(J.mol^− 1^)	1.30	13.63
	K_T_(L.g^− 1^)e^4^	0.28	0.126
	R^2^	0.9592	0.6455
	ARE%	25.10	68.7
	SSE	14.3	843.20
	X^2^	3.98	98.40

**Table 8:** Data of kinetic models.

Kinetic model	Parameters	Investigated polymeric samples	
		PS/PNMANI	PS/PNMANI/CuO
Pseudo first order	k_1_	0.0699	0.1541
	qe	0.2570	5.6790
	R^2^	0.7981	0.6020
Pseudo second order	k_2_	0.4933	0.5521
	qe	1.0172	3.1726
	R^2^	0.9938	0.9971

#### Adsorption isotherms

In general, adsorption isotherms provide information about the adsorption types and how adsorbate molecules or particles are attracted to the surface of adsorbents^79^. The Langmuir isotherm was utilized to model the equilibrium distribution of MO between the aqueous and solid phases. This approach is based on a quantitative assessment of a singular molecular layer forming on the adsorbent’s surface, assuming that no further adsorption layers are developed. The linear relation of Langmuir is represented by Eq. (8)^79^.

8 $$\:\frac{\boldsymbol{c}\boldsymbol{e}}{\boldsymbol{q}\boldsymbol{e}}=\frac{\boldsymbol{c}\boldsymbol{e}}{\boldsymbol{Q}\boldsymbol{m}}+\frac{1}{\boldsymbol{Q}\boldsymbol{m}\boldsymbol{b}}$$

**Fig. 16:**
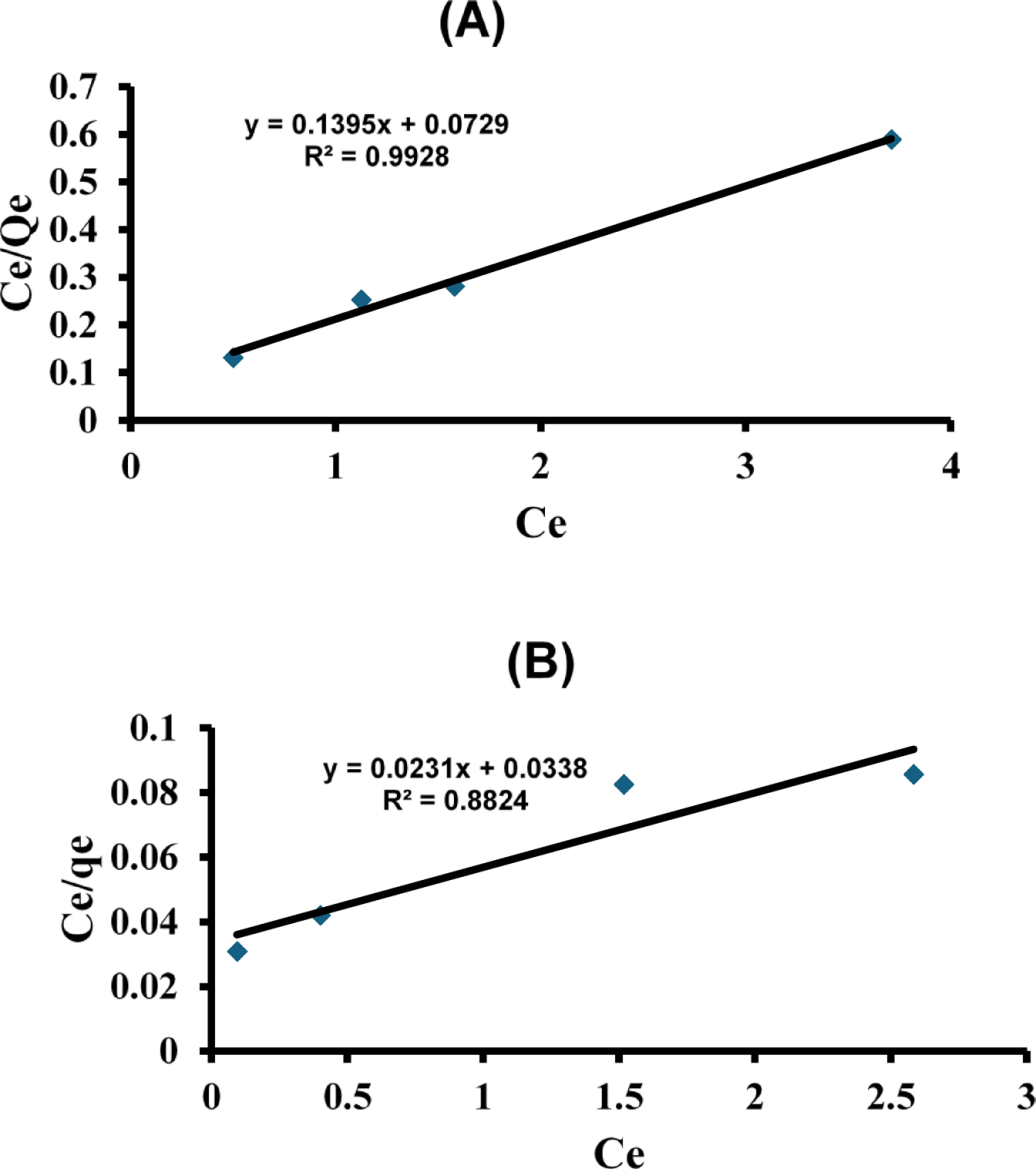
Langmuir isotherm of the investigated samples **(A)** PS/PNMANI and **(B)** PS/PNMANI/CuO.

As Ce represents the equilibrium concentration of the adsorbate (mg L^− 1^), qe is the amount of MO adsorbed per gram of adsorbent at equilibrium (mg g^− 1^), Qm denotes the maximum monolayer adsorption capacity (mg g^− 1^), and b is the Langmuir constant related to adsorption energy. The parameters Qm and b were obtained from the slope and intercept of the Langmuir plot of Ce/qe versus Ce, as shown in Fig. 16.

**Fig. 17:**
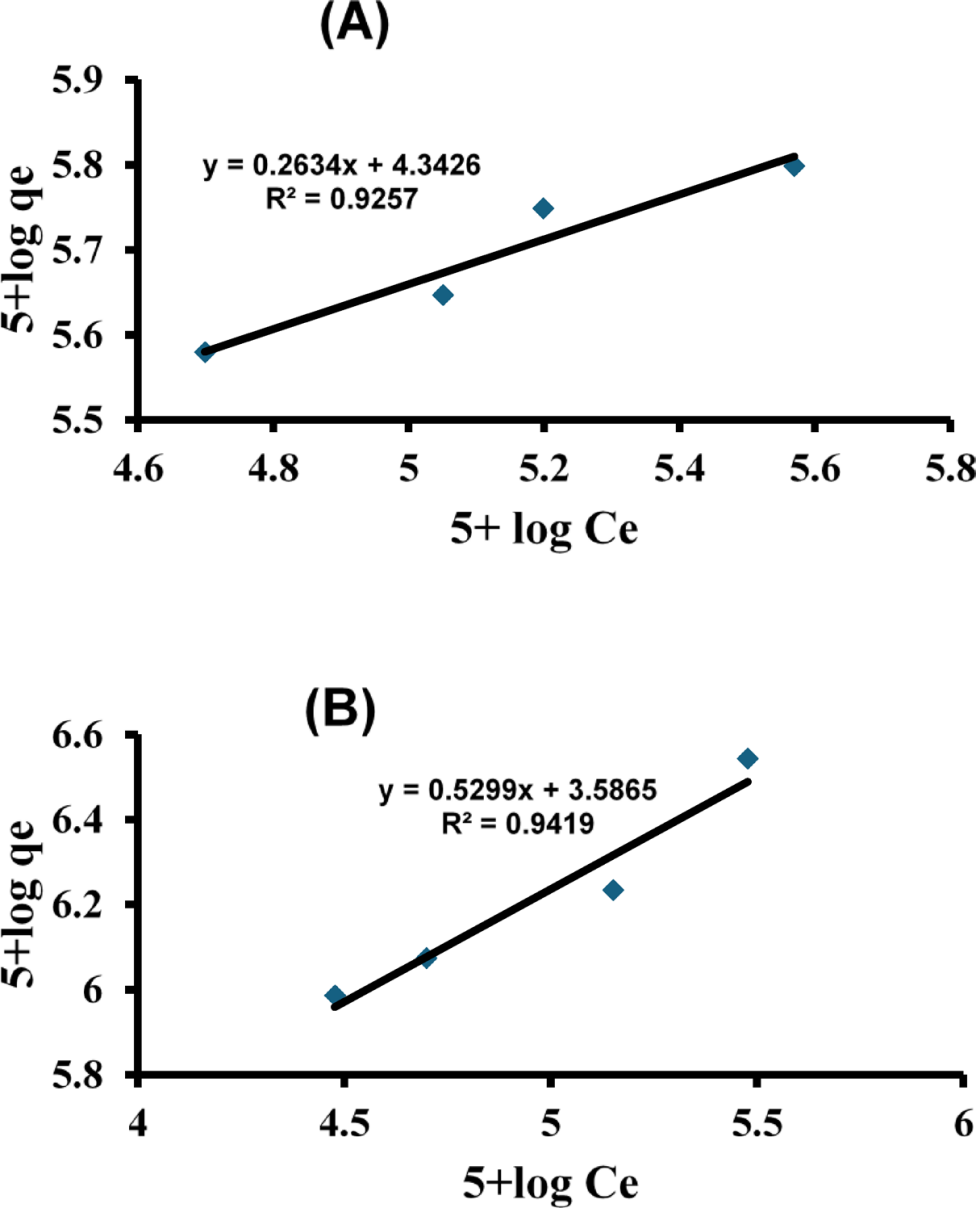
Freundlich isotherm of the investigated samples **(A)** PS/PNMANI and **(B)** PS/PNMANI/CuO.

The Freundlich isotherm^80^ has been widely applied to describe adsorption behavior of different systems. Its linear form (Eq. 9) was used, where qe (mg g^− 1^) represents the amount of MO adsorbed at equilibrium on the copolymer surface, Ce is the equilibrium concentration of MO in solution (mg/L), Kf is the Freundlich constant, and n is the adsorption intensity parameter. Kf indicates the adsorption capacity, while 1/n reflects the adsorption strength. These constants were derived from the slope and intercept of the Freundlich linear plot, as illustrated in Fig. 17.

9 $$\:\mathrm{L}\mathrm{o}\mathrm{g}\:\mathrm{q}\mathrm{e}\hspace{0.17em}=\hspace{0.17em}\mathrm{l}\mathrm{o}\mathrm{g}\:\mathrm{K}_\mathrm{f}+\left(\frac{1}{n}\right)logCe$$

The Temkin isotherm model^81^ incorporates a parameter that accounts for interactions between adsorbed molecules, a feature not considered in the Langmuir model. Thermodynamic analysis indicated that the adsorption of MO onto PS/PNMANI is endothermic, whereas it becomes exothermic when using the composite material.

This model explicitly considers adsorbent–adsorbate interactions, assuming that the heat of adsorption decreases progressively with increasing surface coverage. In contrast to logarithmic variation, and by neglecting very low and very high concentration limits, this decrease is treated as linear with respect to coverage.

10 $$\:\mathrm{q}\mathrm{e}\hspace{0.17em}=\hspace{0.17em}\mathrm{B}\mathrm{T}\:\mathrm{L}\mathrm{n}\:\mathrm{K}\mathrm{T}\hspace{0.17em}+\hspace{0.17em}\mathrm{B}\mathrm{T}\:\mathrm{L}\mathrm{n}\:\mathrm{C}\mathrm{e}$$

**Fig. 18:**
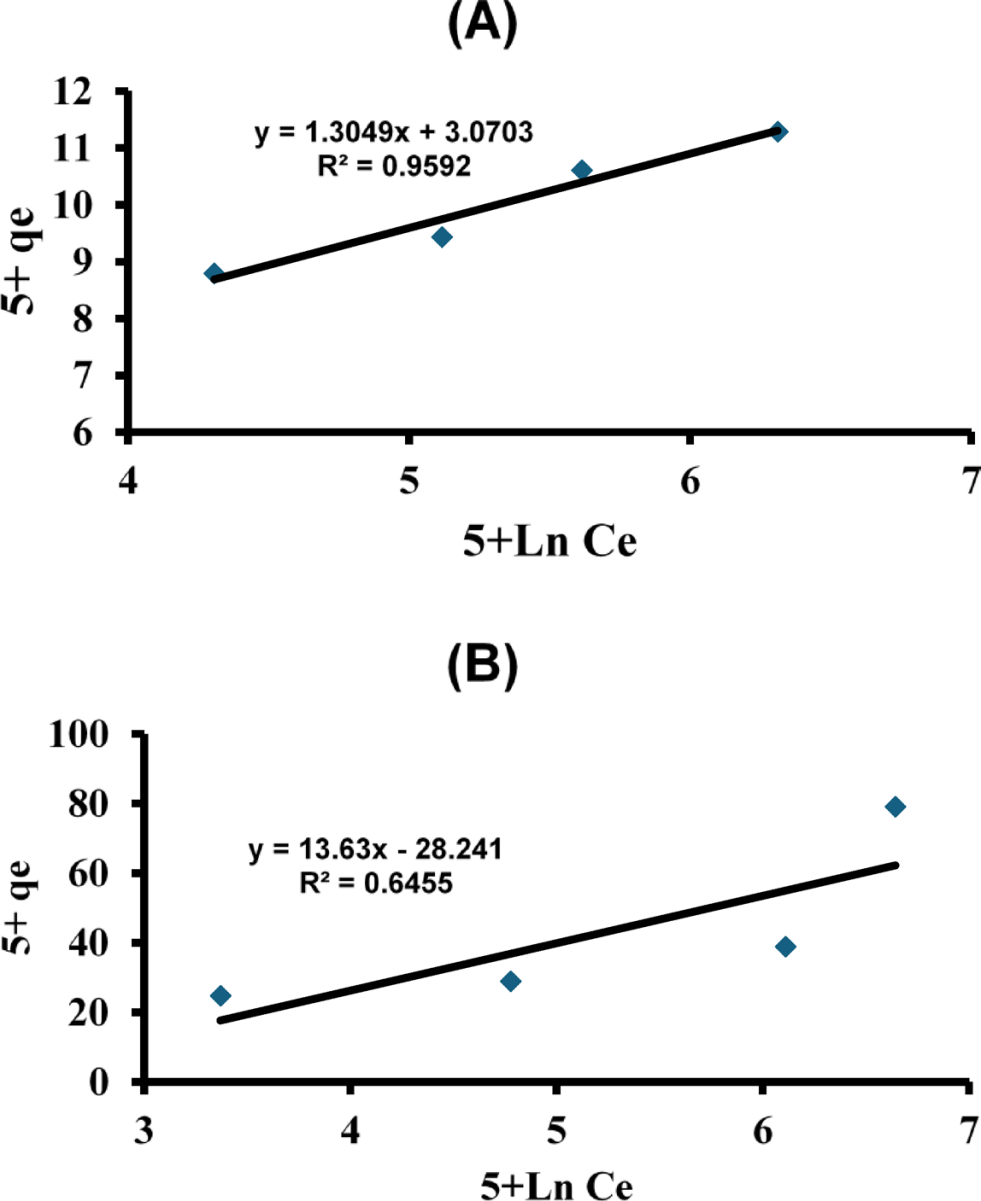
Temkin isotherm of the investigated samples **(A)** PS/PNMANI and **(B)** PS/PNMANI/CuO.

R is the ideal gas constant (8.314 Jmol^−1^K^− 1^). As qe represents MO adsorbed at equilibrium (mg g^− 1^). BT = RT/b is a constant related to the heat of sorption (J mol^−^¹). T is the absolute temperature (K). b is the Temkin isotherm constant and KT is the Temkin equilibrium-binding constant (L g^− 1^). The linear plots of ln(qe) versus ln Ce are presented in Fig. 18. The adsorption data for the three investigated models are summarized in Table 7. From the results, it is evident that MO adsorption on the surface of the studied adsorbents follows a multilayer behavior and is better described by a mixed adsorption model.

#### Regeneration, reusability, sustainability and adsorption confirmation

**Fig. 19:**
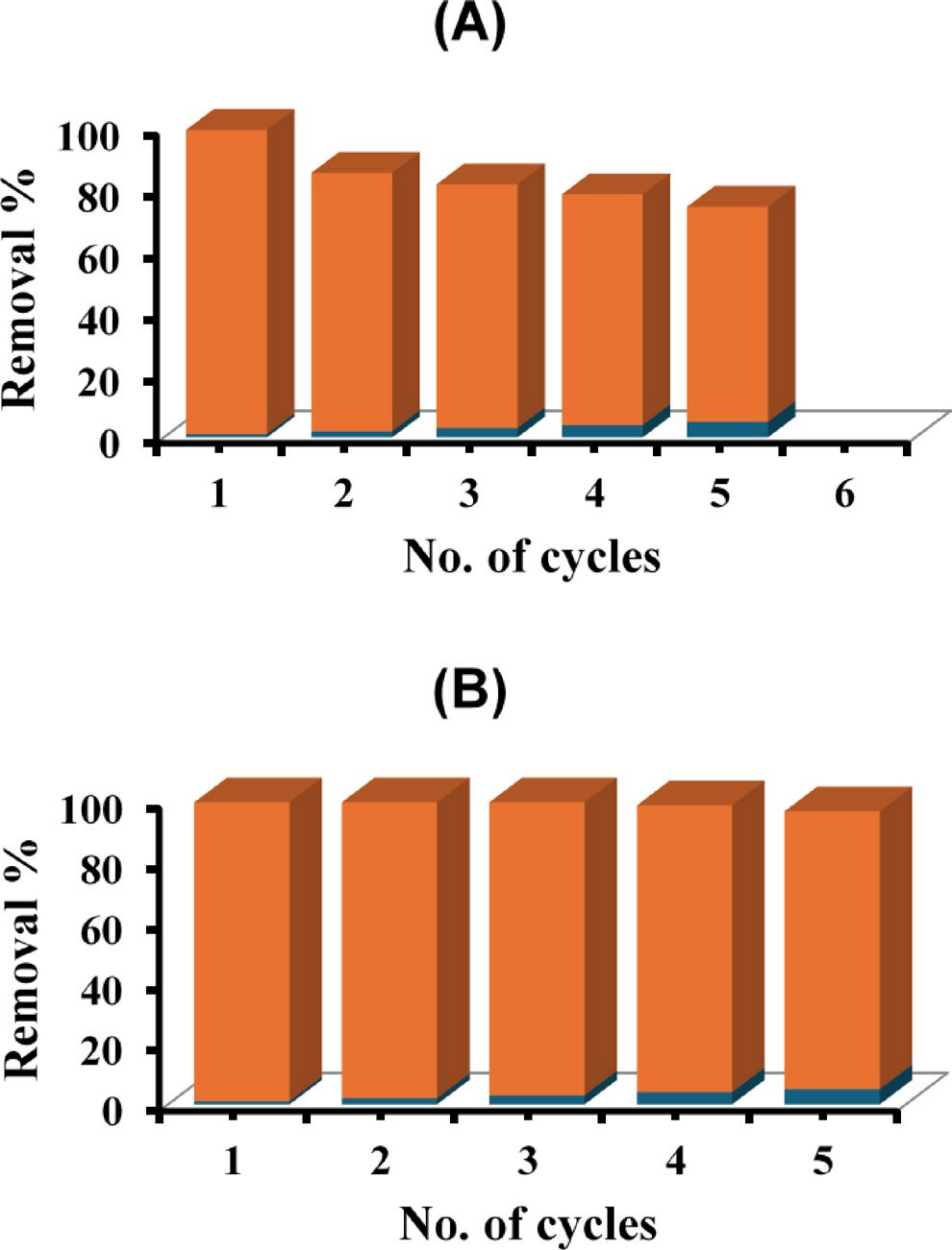
Reusability of both **A)** PS/PNMANI and **(B)** PS/PNMANI/CuO.

The maximum adsorption (Removal %) of MO from treated water sample is 95 and 100% for both PS/PNMANI (0.25 g, 60 min, 298 K and pH = 7) and PS/PNMANI/CuO (0.10 g, 60 min, 298 K and pH = 7) respectively. Both PS/PNMANI and PS/PNMANI/CuO are used as adsorbents for MO from wastewater sample (collected from Beni-Suef station for water treatment) at the same conditions. The removal % still 100% in case of PS/PNMANI/CuO but increase to 99.8% in case of PS/PNMANI with can be attributed to enhancement of adsorption be the soluble anions present in the wastewater. After adsorption both PS/PNMANI and PS/PNMANI/CuO are rinsed in distilled water for 1 h with stirring to desorption of MO (water is a good solvent to MO), washed with methanol then dried at 110 °C till constant weight. The dried loaded samples with MO reused again at the same condition to check the removal%, the above run is repeated 5 cycles and the data are represented in Fig. 19. Which reveal that these samples can be used more than 5 times with still highly efficacy to MO removal 70% in case of PS/PNMANI and 92% in case of PS/PNMANI/CuO. This experiment confirms sustainability and reusability of the present samples especially the composite.

**Fig. 20:**
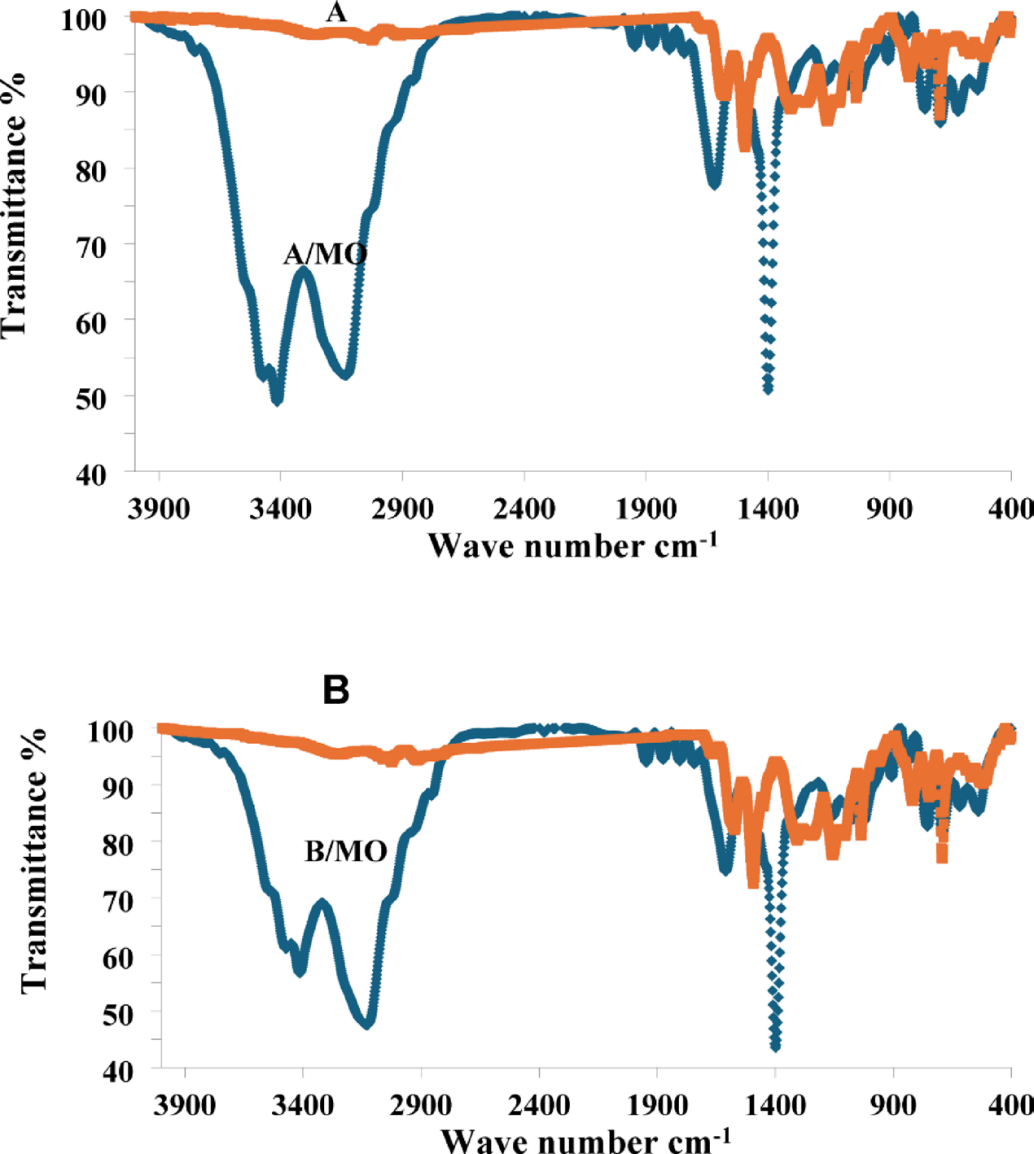
IR spectra for the synthesized polymeric samples **(A)** PS/PNMANI, A/MO) is PS/PNMANI loaded with MO, **(B)** PS/PNMANI/CuO, and B/MO) is PS/PNMANI/CuO loaded with MO.

To confirm the adsorption^82–86^ both PS/PNMANI and PS/PNMANI/CuO loaded with MO are introduced to IR- spectroscopy, the charts compared with unloaded one are present in Fig. 20. The new sharp absorption bands appeared at 1402 cm^−1^in case of both the two samples are attributed to the N-C bond in the MO, this band appears at 1500 cm^− 1^ for unloaded samples. N = N stretching vibration of MO is appeared at 1629 and 1618 cm^− 1^ as sharp bands. In case of unloaded samples appears at 1595 and 1581 cm^− 1^ for PS/PNMANI and PS/PNMANI/CuO respectively. The sharp and broad bands appear at 3167, 3421, 3485 and 3772 cm^− 1^ in case of loaded PS/PNMANI and at 3150, 3423 and 3479 cm^− 1^ in case of loaded PS/PNMANI/CuO are attributed to the SO_3_Na group of MO. These bands confirm the loading of MO on the investigated polymeric samples.

#### Economic analysis

The economic feasibility of this study can be provided as follows:

From the obtained data 0.1 g of composite remove 4.3 mg present in 100 mL wastewater. The approximating price of basic used material in Egypt is 80, 1050, 1500 pound for PS, PNMANI and CuO respectively. From the experimental data (Sect. 2.2.3) 4.0 g of both N-methylaniline and PS and 0.4 g of CuO, the yield of this conversion is 12.5 g due to the absorption of water. The production of 1.0 g of composite costs 0.4256 pound. 1.0 L contains 43 mg MO was purified by 1.0 g composite on reusability of the composite 5 cycles 0.20 g is enough. The final comment is for each 1.0 m^3^ polluted with 10 ppm MO purified by 85.12 pounds. This value is very low compared by activated carbon and/or membranes.

## Conclusion

The PS/PNMANI/CuO composite was successfully fabricated and utilized for the removal of methyl orange from aqueous solutions. The maximum adsorption capacity (Qmax) was found to be 43.29 mg/g, with an optimum removal efficiency of 100% at pH 7 and contact time of 60 min at 298 K using 0.10 g. Compared to the PS/PNMANI blend, the incorporation of CuO enhanced the adsorption performance, highlighting the synergistic effect of CuO nanoparticles in providing additional active sites and improving electrostatic interactions. The synthesis of the present composite is very easy no time or energy consumed. The reusability of these samples give PS/PNMANI and PS/PNMANI/CuO advantage for using in adsorption process as the famous used materials.

## Supplementary Information

Below is the link to the electronic supplementary material.

Supplementary Material 1

## Data Availability

All data supporting the findings of this study are available within the paper.
