# Supplementary material for: Synthesis of facile polystyrene/pol(N-methylaniline)/CuO composite for methyl orange uptake
Source: Sci Rep. 2026 Aug 5;16:24194. doi: 10.1038/s41598-026-63266-9 (PMC13444245; doi:10.1038/s41598-026-63266-9)
Supplement: Supplementary file 1 [file 41598_2026_63266_MOESM1_ESM.docx]

Techno-Economic Study for Small-Scale Textile Wastewater Plant

Techno-Economic Analysis of PS/PNMA/CuO Nanocomposite for Methyl Orange Removal

**Case Study**: 20 m³/day Small Textile Factory, Beni Suef Industrial Zone, Egypt

**1. Process Overview**

Batch adsorption system using 0.1 g/L PS/PNMA/CuO nanocomposite at room temperature 25°C. Contact time 60 min, pH 6.5. Adsorbent regenerated with 0.1M NaOH for 6 cycles.

**2. Design Basis**

- Wastewater flow: 20 m³/day = 4 batches × 5 m³/batch

- Initial MO concentration C₀: 50 ppm

- Final concentration Cₑ: < 5 ppm

- Removal efficiency: 90%

- Adsorbent dose: 1.43 kg/m³ based on q_max_ = 43.3 mg/g

**3. Capital Cost CAPEX - USD**

Item	Specification	Cost USD

Batch reactor SS304	5 m³, mixer, pH control	3,750

Magnetic separator	For CuO recovery	1,350

Storage tanks PE	2 × 5 m³	830

Pumps & piping	2 HP × 2	730

Regeneration tank	1 m³ for NaOH wash	520

Installation 15%	-	1,080

**Total CAPEX**	**8,260 USD**

**4. Operating Cost OPEX - USD/m³**

Cost Item	Consumption	Unit Price	Cost USD/m³

PS/PNMA/CuO adsorbent	0.24 kg/m³	64.5 USD/kg	15.48

Electricity	0.8 kWh/m³	0.10 USD/kWh	0.08

NaOH 0.1M for regeneration	0.05 kg/m³	0.45 USD/kg	0.02

Labor	0.2 h/m³	2.1 USD/h	0.42

Maintenance 3% CAPEX/year	-	-	0.17

Water analysis COD/UV-Vis	-	-	0.31

Total OPEX	16.48 USD/m³

**5. Economic Indicators**

- Annual treatment cost: 16.48 × 20 × 300 days = 98,880 USD/year

- Cost vs Activated Carbon: 250 USD/m³ → 93.4% reduction .

- Payback vs Environmental fine: < 7 days

- NPV 5 years @ 10% discount: +1.2 Million USD if avoiding fines

**6. Conclusion**

PS/PNMA/CuO shows strong economic viability for SMEs in Egypt due to low material cost, room temperature operation, and 6-cycle regeneration. CAPEX recovered in <1 week compared to regulatory penalties.
